# *Agrobacterium tumefaciens*-Mediated Transformation of the Lichen Fungus, *Umbilicaria muehlenbergii*

**DOI:** 10.1371/journal.pone.0083896

**Published:** 2013-12-30

**Authors:** Sook-Young Park, Min-Hye Jeong, Hai-Ying Wang, Jung A. Kim, Nan-Hee Yu, Sungbeom Kim, Yong Hwa Cheong, Seogchan Kang, Yong-Hwan Lee, Jae-Seoun Hur

**Affiliations:** 1 Korean Lichen Research Institute, Sunchon National University, Sunchon, Korea; 2 Dept. of Agricultural Biotechnology, Fungal Bioinformatics Laboratory, Center for Fungal Genetic Resources, and Center for Fungal Pathogenesis, Seoul National University, Seoul, Korea; 3 Dept. of Biology, Sunchon National University, Sunchon, Korea; 4 College of Life Sciences, Shandong Normal University, Jinan, China; 5 Dept. of Plant Pathology & Environmental Microbiology, The Pennsylvania State University, University Park, Pennsylvania, United States of America; Soonchunhyang University, Republic of Korea

## Abstract

Transformation-mediated mutagenesis in both targeted and random manners has been widely applied to decipher gene function in diverse fungi. However, a transformation system has not yet been established for lichen fungi, severely limiting our ability to study their biology and mechanism underpinning symbiosis via gene manipulation. Here, we report the first successful transformation of the lichen fungus, *Umbilicaria muehlenbergii*, via the use of *Agrobacterium tumefaciens*. We generated a total of 918 transformants employing a binary vector that carries the hygromycin B phosphotransferase gene as a selection marker and the enhanced green fluorescent protein gene for labeling transformants. Randomly selected transformants appeared mitotically stable, based on their maintenance of hygromycin B resistance after five generations of growth without selection. Genomic Southern blot showed that 88% of 784 transformants contained a single T-DNA insert in their genome. A number of putative mutants affected in colony color, size, and/or morphology were found among these transformants, supporting the utility of *Agrobacterium tumefaciens*-mediated transformation (ATMT) for random insertional mutagenesis of *U*. *muehlenbergii*. This ATMT approach potentially offers a systematic gene functional study with genome sequences of *U. muehlenbergii* that is currently underway.

## Introduction

Availability of an efficient transformation system is crucial for the experimental study of gene function. Polyethylene glycol (PEG)-mediated introduction of DNA to protoplasts has been widely utilized for fungal transformation. Generation of good protoplasts through efficient digestion of the fungal cell wall using a mixture of hydrolytic enzymes is critical for high transformation efficiency [1]–[3]. Accordingly, this approach is not readily applicable to fungi that are recalcitrant to cell wall degrading enzymes.

*Agrobacterium tumefaciens* has been found to introduce and integrate engineered T-DNA efficiently into the genome of *Saccharomyces cerevisiae* [4] and several filamentous fungi [5]. *A. tumefaciens*-mediated transformation (ATMT) works well with several intact fungal tissues (e.g., spores, mycelia, gill tissues from mushroom), offering an alternative means for transforming fungi that do not readily produce protoplasts. In addition, the transformation efficiency of *Aspergillus awamori* via ATMT was up to 600-fold higher than by PEG-mediated transformation [5]. Since these initial reports of fungal ATMT, this method has been successfully applied to transform phylogenetically diverse fungi. Many binary vectors with different features and utilities have been developed to support molecular genetic studies of fungi via ATMT [6]–[9]. Random insertional mutagenesis of the fungal genome via ATMT has been successfully applied to several fungi to identify many genes essential for their life cycle and pathogenicity [8], [10]–[13]. ATMT also facilitates efficient targeted gene manipulation via homologous recombination [4], [14], [15].

More than 19% of all known fungi form lichens [16], [17], and nearly 98% of them belong to the phylum Ascomycota. Lichens are mutualistic organisms producing a myriad of potentially useful metabolites [18] that exhibit antitumor [19]–[21], antimicrobial [22]–[38], anti-inflammatory [39], [40], and antioxidant activities [30]–[32], [40]. Although lichens consist of a fungal partner (the mycobioint) and a photosynthetic alga, cyanobacterium, or both (the photobiont) [41], it is generally thought that most secondary metabolites of lichens are produced by the mycobioint [38], [42], [43]. Heterologous expression of genes of lichen fungi in surrogate fungi such as *Aspergillus* species has been evaluated [44], [45]. This heterologous expression offers an alternative approach to explore lichen polyketide biosynthesis. However, this approach still remains to be validated the production of identical substances that generated by lichen thallus.

**Figure 1:**
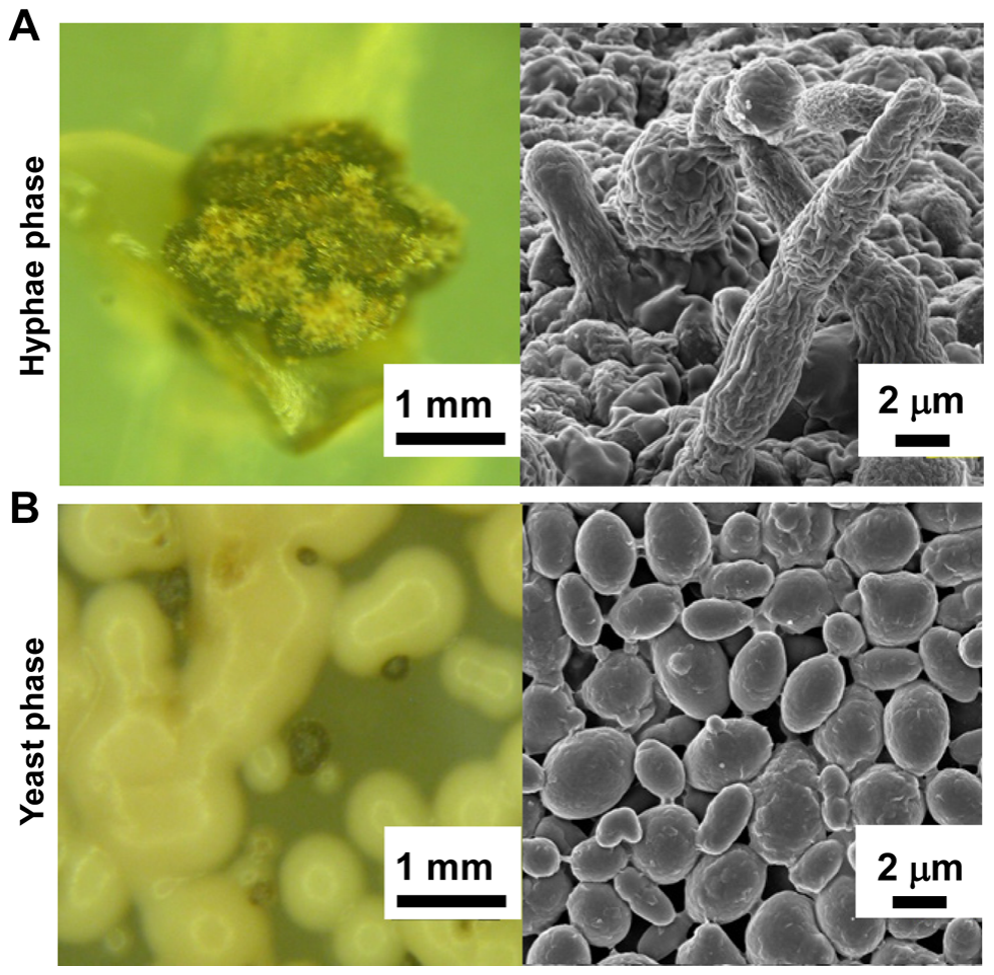
Morphologies of *U. muehlenbergii*. Images of the hyphae phase (A) and the yeast phase (B) were obtained using a stereoscope (left) and a scanning electron microscope (right).

With efficient transformation methods, direct mutagenesis of candidate genes in lichen fungi via random insertional mutagenesis or targeted deletion offers a more direct approach to discover and study such genes. Unfortunately, however, no such method has been established in lichen fungi. This is mainly because axenically cultured lichen fungi typically grow extremely slowly and have a tough cell well [46], [47]. Recently, we found a relatively fast growing and dimorphic lichen fungus, *Umbilicaria muehlenbergii* (Figure 1). Relative to other lichen fungi, *U*. *muehlenbergii* could be grown easily and quickly in liquid nutrient media. Moreover, polysaccharides from *Umbilicaria* species showed antitumor and anti-HIV activities [48]–[50]. Establishment of a transformation system for *U*. *muehlenbergii* will offer a valuable tool for studying its genes as well as those in other lichen fungi. We therefore tested whether ATMT could be applied to *U. muehlenbergii*, this has led to the generation of mitotically stable transformants. To our knowledge, this is the first time transformation of a lichen fungus is reported. This method may be applicable to other lichen fungi, potentially enabling direct manipulations of their genes and genomes.

## Results

### Isolation of *U. muehelenbergii*

Lichen thalli of *U. muehlenbergii* were collected from a rock at Mt. Tulaopoding, Jilin province, China in 2012. The lichen was identified by Dr. Wang in Shandong Normal University (Jinan 250014, China). A voucher specimen was deposited in the herbarium of the university and duplicated in the Korean Lichen Research Institute (KoLRI) at Sunchon National University (Sunchon 540–950, Korea). Lichen-forming fungus (LFF) of *U. muehlenbergii* was isolated by the tissue culture method [51] from the lichen thalli. Hyphal and yeast-like growth forms of the LFF were isolated. A number of conidia were produced both on solid PDA medium and in liquid PDB medium. Analysis of internal transcribed spacer (ITS) sequence indicated that the two growth forms of LFF were genetically identical to the originally isolated *U. muehlenbergii* strain.

### *Agrobacterium Tumefaciens*-mediated Transformation of *U. muehlenbergii*

**Figure 2:**
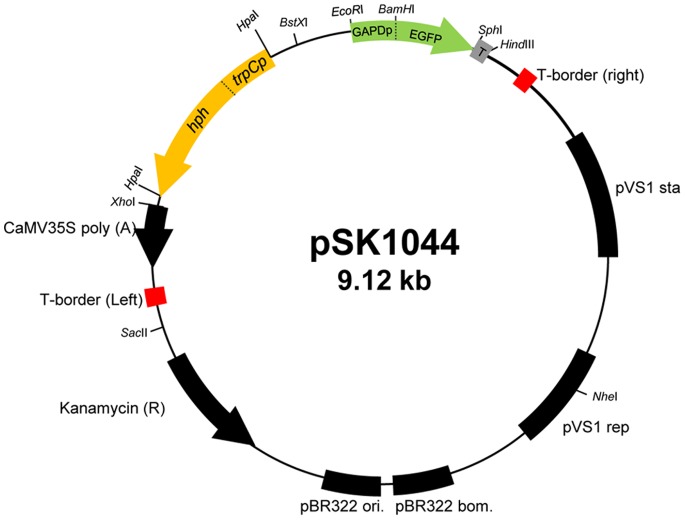
Map of binary vector pSK1044. pBHt2 contains the hygromycin B resistance gene under the control of the *Aspergillus nidulans trp*C promoter [8] was used to construct pSK1044 by insertion of the *eGFP* gene cassette between the *Eco*RI and *Hin*dIII sites in the multi-cloning site of pBHt2.

**Figure 3:**
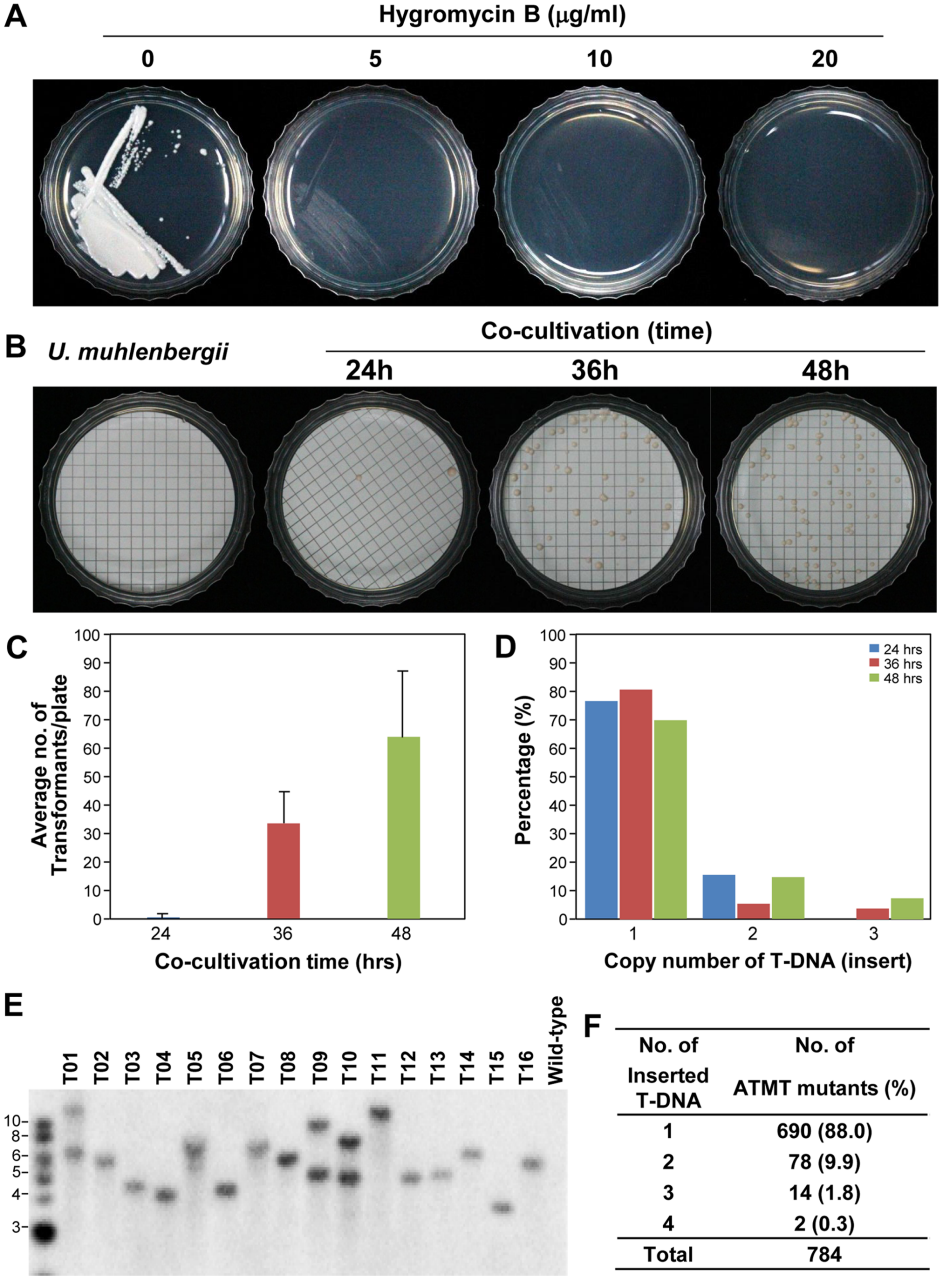
Growth, transformation efficiency, and Southern analysis. *(*A) Sensitivity of a wild-type *U. muehlenbergii* strain to hygromycin B was evaluated by culturing it on PDA amended with 0, 5, 10 and 20 µg/ml of hygromycin B. (B) Membranes overlaid with only fungal cells (1^st^ on the left), and a mix of *A. tumefaciens* and fungal cells (2^nd^, 3^rd^ and 4^th^) were placed on PDA containing 20 µg/ml hygromycin B after culturing on co-cultivation medium for 24, 36 and 48 hrs (2^nd^, 3^rd^ and 4^th^). The pictures were taken after 28 days of culture on this selection medium. (C) Effect on the transformation efficiency by increasing the co-cultivation time. Data presented as the average of eight plates per treatments. Error bars indicates standard error. (D) Distribution of T-DNA copy number among transformants of different time interval of co-cultivation. Genomic DNAs were digested with *Hin*dIII, a restriction enzyme that does not cut the *hph* cassette (see Figure 2). The digested DNAs, fractionated using 0.7% agarose gel and transferred to a nylon membrane, were probed with a labeled *hph* cassette. (E) Representative result of Southern blot analysis from 15 randomly selected transformants and a wild-type strain. (F) Distribution of T-DNA copy numbers among 784 transformants.

In order to test whether *A. tumefaciens* can transfer T-DNA to *U. muehlenbergii* (Figure 1), we used the binary vector pSK1044 (Figure 2 and Information S1). First, we evaluated the sensitivity of *U. muehlenbergii* to hygromycin B to determine an optimal concentration for selecting transformants. The fungus was highly sensitive to hygromycin B, with complete growth inhibition at 20 µg/ml (Figure 3A). Therefore this concentration was used for selecting transformants.

Co-cultivation of *A. tumefaciens* cells with yeast-like conidia of *U. muehlenbergii* in the presence of acetosyringone (AS), a phenolic compound that is secreted from wounded plants, led to the appearance of hygromycin B-resistant colonies approximately one month after transfer to selection medium. To test the effect of co-cultivation time on transformation efficiency, we evaluated 24, 36, and 48 hrs of co-cultivation. The longer co-cultivation at 48 hrs led to >64-fold increase in the number of transformants, resulting in 27 to 98 transformants per 1×10^6^ conidia (Figure 3B and 3C). However, increasing the co-cultivation time decreased the frequency of transformants that contained a single copy of T-DNA (Figure 3D). Therefore, we choose 36 hrs of co-cultivation time to generate transformants for *U. muehlenbergii*.

Assessment of the mitotic stability of 24 transformants showed that all maintained hygromycin B resistance after being cultured for five generations in the absence of hygromycin B (data not shown).

### Generation of a Pool of T-DNA Tagged Mutants and Expression of Green Fluorescent Protein in *U. muehlenbergii*

We conducted insertional mutagenesis of *U. muehlenbergii* via ATMT, resulting in 918 hygromycin B-resistant mutants. Southern analysis of 784 transformants revealed that 88.0% (690 transformants) appeared to have a single copy T-DNA insertion (Figure 3E and 3F). Small fractions of transformants showed two (9.9%), three (1.8%), and four or more (0.3%) copies of T-DNA inserted in their genome (Figure 3F).

**Figure 4:**
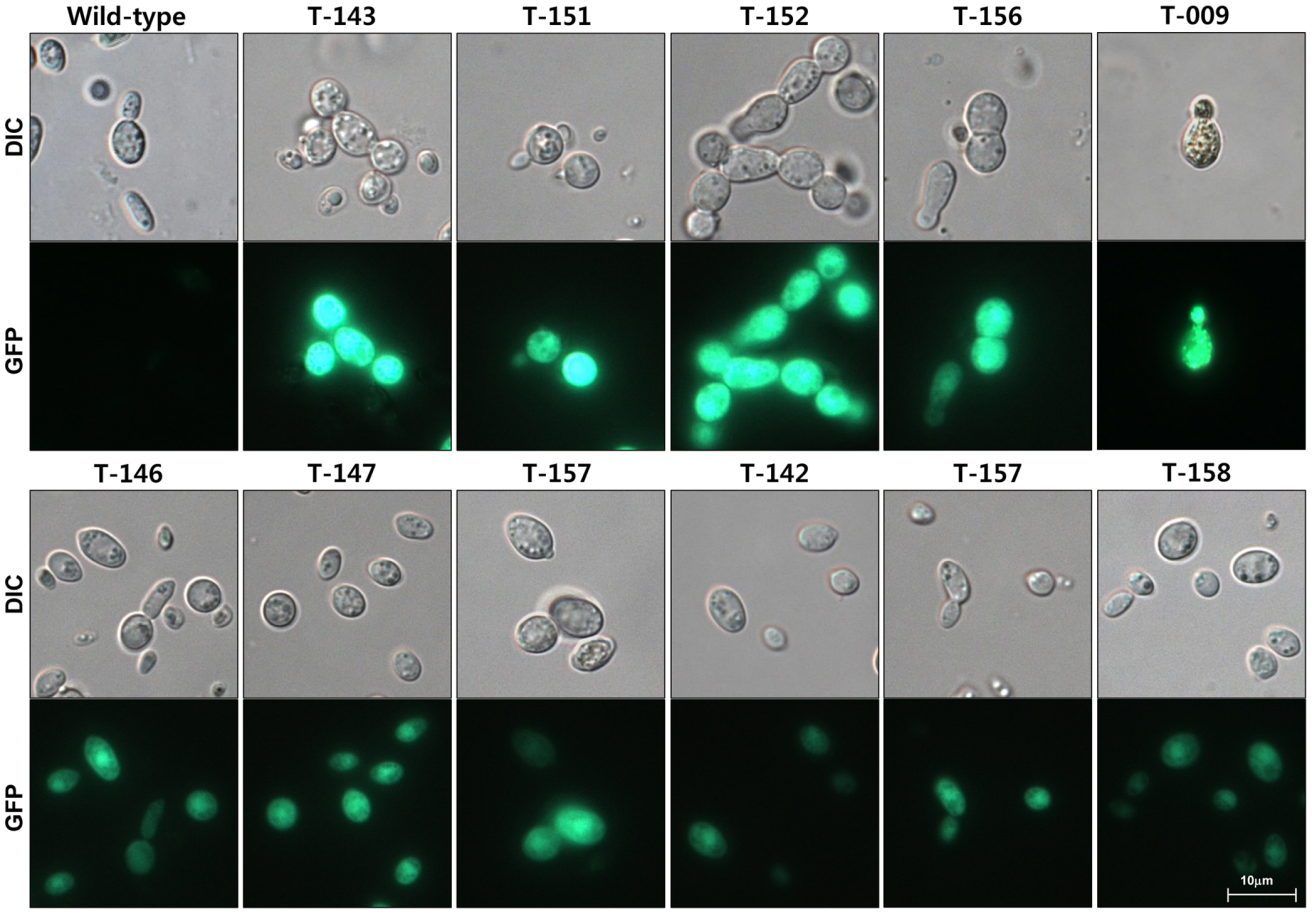
Expression of eGFP in 11 transformants. DIC, differential interference contrast images; GFP, fluorescence image.

We also tested the transformants for green fluorescence, as pSK1044 contains the *eGFP* gene under the control of *Cochliobolus heterostrophus GAPD* gene promoter in its T-DNA. Thirteen randomly selected transformants were screened using fluorescence microscopy (Table 1). All of them consistently displayed green fluorescence, although fluorescence in five transformants, including UmT-009, UmT-143, UmT-151, UmT-152 and UmT-156, was stronger than in the others (Figure 4). Differential expression of *eGFP* among these transformants suggested that the level of *eGFP* expression might vary depending on the sites of T-DNA integration (i.e., positional effect).

**Table 1:** Results from BlastX searches of the NCBI database with *U*. *muehlenbergii* genomic sequences flanking inserted T-DNA as queries.

Transformant	No. ofT-DNA inserted	Best BlastX match	E-value
UmT-001	1	No match	
UmT-002	1	No match	
UmT-003	1	No match	
UmT-004	1	No match	
UmT-005	2	No match	
UmT-008	1	(EKD20173.1) Fungal Zn binuclear cluster domain [*Marssonina brunnea* f. sp. ‘multigermtubi’MB m1]	3E-07
UmT-009	1	No match	–
UmT-010	1	(EKD17225.1) hypothetical protein MBM 04802 [*Marssonina brunnea* f. sp. ‘multigermtubi’ MB m1]	5E-25
UmT-011	1	(EFX04015.1) mynd domain containing protein [*Grosmannia clavigera* kw1407]	0.011
UmT-012	1	No match	
UmT-013	1	No match	
UmT-014	1	No match	
UmT-016	3	No match	
UmT-017	1	No match	
UmT-018	1	No match	
UmT-020	1	(EKD12163.1) contractile ring protein lmp2 [*Marssonina brunnea* f. sp. ‘multigermtubi’ MB m1]	1E-24
UmT-141	1	No match	
UmT-142	1	No match	
UmT-143	1	No match	
UmT-146	1	No match	
UmT-147	1	No match	
UmT-148	1	(ERF68946.1) hypothetical protein EPUS_08180 [*Endocarpon pusillum* Z07020]	3E-38
UmT-151	2	(CCD44366.1) similar to adenosine deaminase family protein [*Botryotinia fuckeliana* T4]	5E-36
UmT-152	1	No match	
UmT-153	2	No match	
UmT-156	1	(EHY52878.1) putative snf7 family protein [*Neofusicoccum parvum* UCRNP2]	1E-28
UmT-157	1	No match	
UmT-158	2	(EKV12695.1) conserved hypothetical protein [*Paracoccidioides brasiliensis* Pb18]	1E-13
UmT-160	1	(EKG14035.1) Phospholipase D/Transphosphatidylase [*Macrophomina phaseolina* MS6]	4E-09
UmT-161	1	No match	
UmT-162	1	(ELR06043.1) hypothetical protein GMDG 07754 [*Geomyces destructans* 20631-21]	1E-24
UmT-163	1	No match	
UmT-164	1	No match	
UmT-165	2	(EME83976.1) hypothetical protein MYCFIDRAFT 210745 [*Pseudocercospora fijiensis* CIRAD86]	1E-90
UmT-166	1	(EQB57531.1) cytochrome P450 [*Colletotrichum gloeosporioides* Cg-14]	1E-04
UmT-167	1	No match	
UmT-168	1	(XP002845815.1) histone deacetylase phd1 [*Arthroderma otae* CBS 113480]	8E-138
UmT-169	1	No match	
UmT-170	1	(EPE28228.1) LDH C-terminal [*Glarea lozoyensis* ATCC 20868]	2E-21
UmT-261	1	(XP003300732.1) hypothetical protein PTT12065 [*Pyrenophora teres* f. sp. *teres* 0–1]	5E-09
UmT-262	1	(EGF81700.1) hypothetical protein BATDEDRAFT 87213 [*Batrachochytrium dendrobatidis* JAM81]	6.9E-01
UmT-263	1	No match	–
UmT-264	1	(CAK44308.1) unnamed protein product [*Aspergillus niger*]	2E-20
UmT-265	1	No match	
UmT-266	1	(XP001629411.1) predicted protein [*Nematostella vectensis*]	2E-02
UmT-267	1	(XP002152012.1) C2H2 transcription factor (RfeC) [*Talaromyces marneffei* ATCC18224]	4E-16
UmT-269	1	(XP001549393.1) hypothetical protein BC1G 12121 [*Botryotinia fuckeliana* B05.10)	3E-20
UmT-270	1	No match	–
UmT-272	1	No match	–
UmT-400	1	No match	–

### Identification of Genomic Sequences Flanking Inserted T-DNA

**Figure 5:**
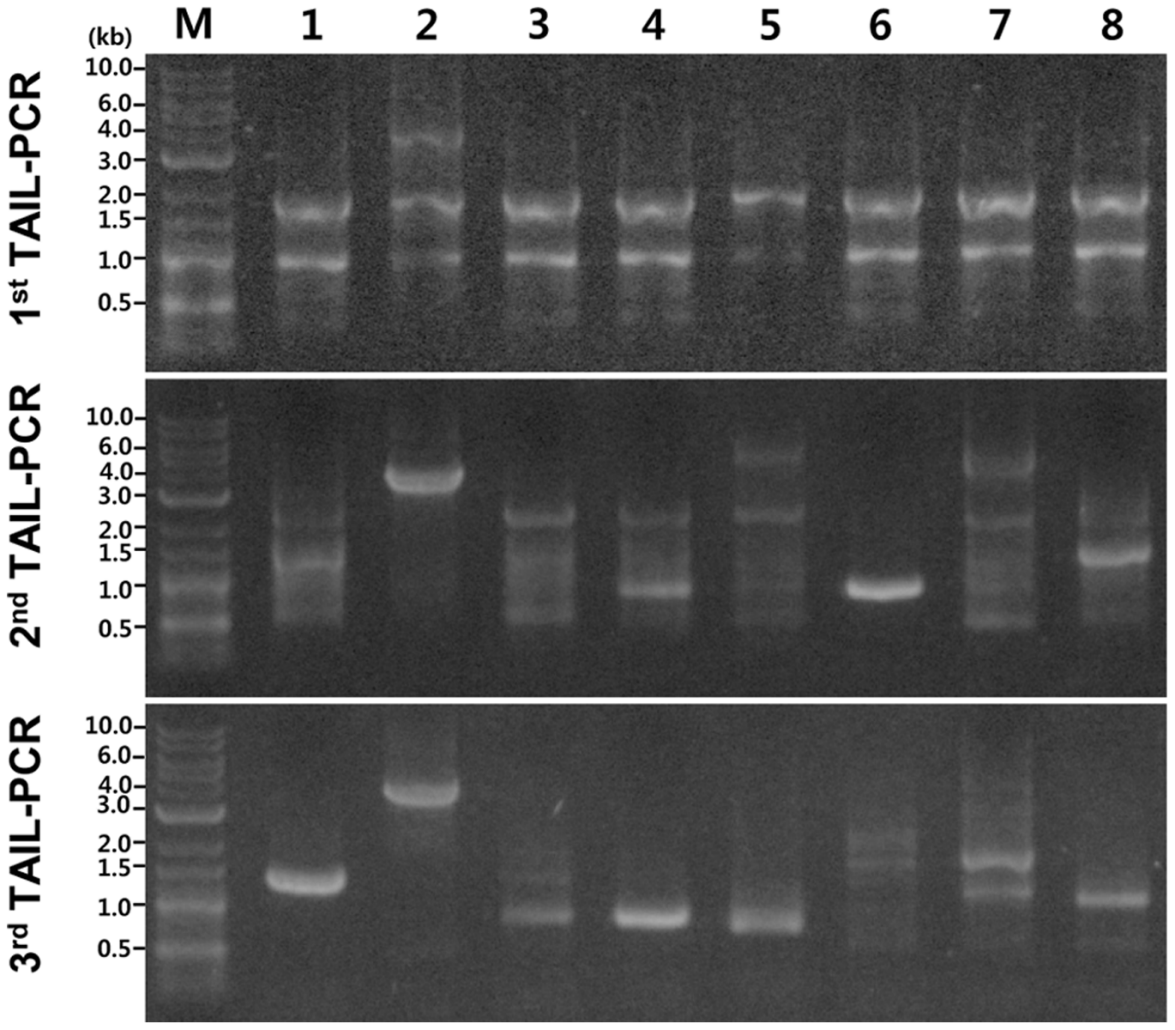
Agarose gel analysis of products resulted from TAIL-PCR of eight randomly selected transformants. The gels from the top to the bottom show products from the primary, secondary, and tertiary reactions, respectively.

**Figure 6:**
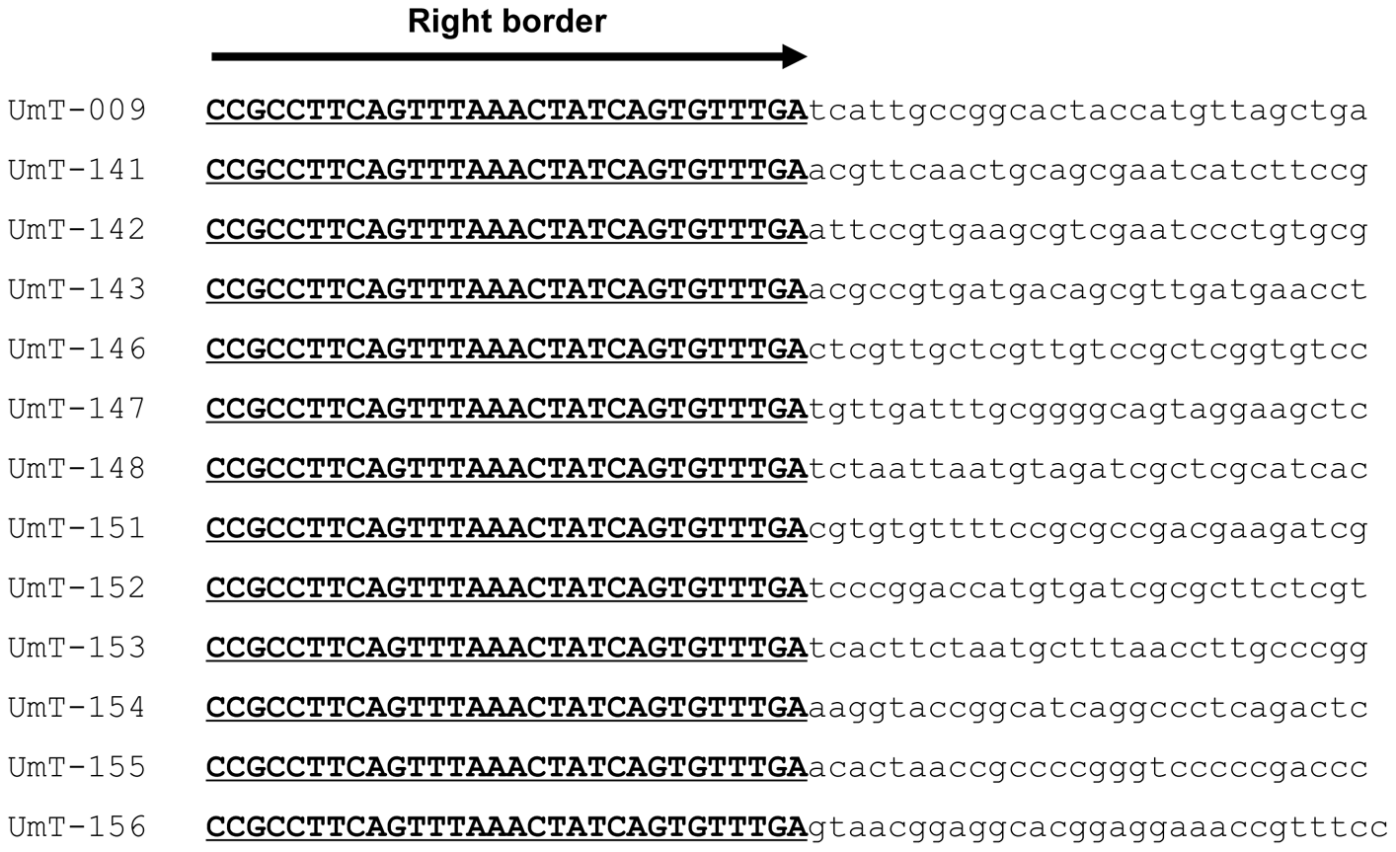
Genomic sequences of *U. muehlenbergii* flanking the right border of inserted T-DNA. All sequences are shown 5′→3′. Bold letters correspond to the 30 bp border sequences.

Thermal asymmetry interlaced-PCR (TAIL-PCR) and subsequent sequencing were used to identify the genomic locations of T-DNA insertions and the context of flanking sequences in selected transformants. To increase the efficiency of TAIL-PCR in amplifying the flanking regions, we employed a mix of arbitrary degenerate primers (AD-1,2,3,4, and 6 = 1∶1:1∶1:1) with one specific primer for the primary (RB1), secondary (RB2-1) and tertiary (RB3) PCR reactions. This approach led to successful amplification of genomic sequences flanking the right border of the inserted T-DNA from all 50 transformants analyzed (Figure 5, Table 1). The size of the PCR products ranged from 0.2 kb to 4 kb. Products from primary PCR reactions typically displayed similar band patterns, whereas more specific bands appeared in secondary and tertiary PCR reactions (Figure 5). Sequence comparison among 50 flanking regions (Information S2) suggests that insertion of T-DNA appears to be without preferential sequence contexts (Figure 6 and Information S2). Unlike in plants or other fungi tested [52]–[55], we did not find any truncated T-DNA in these 50 transformants (Figure 6 and Information S2).

Since the genome of *U. muehlenbergii* has not yet been sequenced, sequences of individual flanking regions were used to search the GenBank protein database using BLASTX. No significant match was found with sequences from 30 transformants, whereas flanking sequences from the remaining 20 transformants matched sequences corresponding to known or hypothetical gene products (Table 1).

### Phenotypic Characterization of Transformants

**Figure 7:**
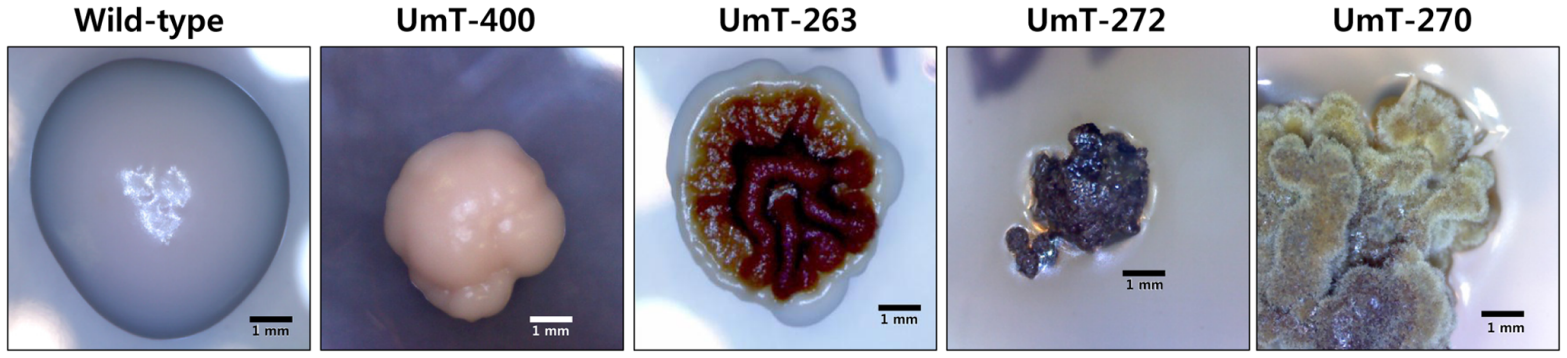
Colonies of four putative mutants of *U. muehlenbergii* generated via ATMT. From left to right, colonies of a wild-type strain and four mutants are shown. These strains were grown for 28 days on PDA.

Phenotypic screening of transformants led to the identification of putative mutants that differ from the wild-type strain in colony color, size, and/or morphology (Figure 7). Compared with the round, white colonies of the wild-type *U. muehlenbergii* typically formed on PDA, colonies of some transformants displayed differential morphological features including heavily pigmented (UmT-263 and UmT-272), reduced size (UmT-400 and UmT-272), and/or different morphologies (UmT-263, UmT-272 and UmT-270) (Figure 7). One putative mutant, UmT-270, exhibited pseudohyphal growth with yellow pigmentation (Figure 7). However, sequences flanking T-DNA in these putative mutants did not exhibit significant similarities to any previously known gene products in the GenBank database.

## Discussion

Lichens have colonized a wide range of ecosystems, including extreme environments such as deserts and arctic regions. As summarized in the Introduction, many lichens are known to produce pharmacologically active metabolites [18]–[40]. Lack of a transformation system for lichen fungi has hampered efforts to study the genetic mechanisms underpinning important biological and ecological processes associated with lichens and their mycobionts. Success in ATMT of diverse fungi in both the Ascomycota and the Basidiomycota [5], [8], [56]–[58] motivated us to apply ATMT to *U. muehlenbergii*. Successful transformation of *U. muehlenbergii* now makes it possible to manipulate its genes and also opens the possibility of using this fungus as a surrogate (i.e., heterologous expression system) for studying the function of genes from other fungi.

The observed efficiency of transformation, 27 to 98 transformants per 1×10^6^ conidia, is not very high compared to those of other fungi [5], [8], [48]. Several studies showed that the efficiency of fungal ATMT is affected by several factors, including a) the concentration of acetosyringone (AS), b) *A. tumefaciens* strain and its growth conditions, c) numbers of fungal and bacterial cells mixed for co-cultivation [4], [5], [8], [10], [57]. Since AS functions as a signal for activating a set of virulence (*vir*) genes in *A. tumefaciens* that are required for T-DNA transfer [59], the presence of AS is essential for ATMT of fungi [5], [8], [57]. Adjustments of AS concentration during the induction and co-cultivation stages may help improve the efficiency. The choice of an *A. tumefaciens* strain is another factor that can be explored, as some strains of *A. tumefaciens* worked better than others in transforming fungi (S. Kang, unpublished result). In addition, the growth conditions of *A. tumefaciens* cells prior to co-cultivation also affected transformation efficiency [8], [48], potentially offering another control point that can be improved and the ratio of bacterial to fungal cells can also be adjusted optimally.

Since many of these factors may also affect the number of T-DNA copies inserted in individual transformants [8], [13], [57], simply employing conditions that provide the greatest number of transformants may not be desirable for certain applications, such as insertional mutagenesis and targeted gene disruption. A key advantage of insertional mutagenesis via ATMT over chemical or radiation mutagenesis is that inserted T-DNA serves as a convenient tag for cloning and identifying mutated genes (e.g., Figure 6 and Table 1). Insertion of T-DNA at multiple loci in a mutant, therefore, can cause a substantial nuisance when it comes to identifying the gene causing a phenotype of interest. For such mutants from fungi with a sexual stage, a genetic cross can be performed to determine which T-DNA segregates with the mutant phenotype before cloning the mutated gene. However, for fungi like *U. muehlenbergii* that require a relatively long time to complete the sexual cycle, genomic sequences flanking all T-DNA inserts need to be functionally validated (e.g., complementation, targeted mutation or both) to characterize the gene of interest. Thus, for efficient insertional mutagenesis and subsequent functional analyses of mutated genes, employing transformation conditions that ensure a high percentage of transformants possessing a single copy of T-DNA is highly desirable. Fortunately, with 36 hrs of co-cultivation, only one copy of T-DNA was inserted in the genome of 88% of the *U. muehlenbergii* transformants (Figure 3E), which is higher than in other fungi [5], [57], [60].

Isolation of three different types of putative morphological mutants from a pool of 918 transformants supports the utility of ATMT for mutagenizing *U. muehlenbergii* via random insertion of T-DNA and subsequently identifying the mutated genes (Figure 7). Pseudohyphal growth of UmT-270 (Figure 7) suggests that a gene required for the regulation of a dimorphic switch may be disrupted. Identification of this gene as well as those tagged in other mutants will help understand how growth is controlled in *U. muehlenbergii*. Unfortunately, amplified genomic sequences that flank the right border of T-DNA in these mutants did not exhibit significant similarity to any known or hypothetical gene products (Table 1). It is possible that the genes tagged in these mutants may be unique to *U. muehlenbergii*. Alternatively, T-DNA insertion may be more indirectly, e.g. by inactivating a promoter or destabilizing mRNA.

In addition, to examine homologous recombination, we conducted amplification of four conserved MAP kinase genes in eukaryotes [61], *Ste7*, *Fus3*, *Hog1*, and *Ste12*, with degenerated primer sets to acquire sequence information of the genes. However, no amplified fragment was obtained from *Ste7* and *Ste12* and no sequence matches were obtained for *Fus3* and *Hog1*, suggesting that the orthologs in *U. muehlenbergii* may have unique start and/or end sequences. Therefore, genome sequence of *U. muehlenbergii* will be needed to carry out homologous recombination.

Since genome sequencing of *U. muehlenbergii* is currently underway, we will integrate T-DNA insertion mutants and phenotype data into *U. muehlenbergii* genome data. In addition, we will apply this system for homologous recombination of the *U. muehlenbergii* and other lichen-forming fungi. This ATMT system will open an era of systematic gene functional studies for lichen fungi.

## Materials and Methods

### Fungal Cultures and Growth Conditions

A culture of *U. muehlenbergii* KoLRI_LF000956, obtained from the Korean Lichen Center at Sunchon National University (Sunchon 540–742, Korea), was stored in the form of a conidial suspension in 15% glycerol at –80°C. It was revitalized by shake culturing in 100 ml of potato dextrose broth (PDB, Difco Laboratories) at 150 rpm and 15°C in the dark. For transformation, after harvesting yeast-like cells in 14 days old culture were harvested by centrifugation at 5,000 rpm for 5 min and washed three times with sterilized distilled water to remove polysaccharides.

### Amplification of ITS Region and Sequencing

The ITS region was amplified from the two growth forms of LFF and the thallus of *U. muehlenbergii* with the primers ITS4 and ITS5 (Table 2) using i-StarMAXII PCR master mix system (iNtRON Biotechnology, Sungnam city, Korea). A Takara PCR thermal cycler MP (Takara, Tokyo, Japan) was employed for 30 cycles of PCR reaction. The amplified PCR products were purified using MEGAquick-spin Total Fragment DNA Purification Kit (iNtRON Biotechnology) and sequenced on both strands with the same primers used for the PCR amplification. ITS sequences were analyzed through NCBI (http://www.ncbi.nlm.nih.gov/).

**Table 2:** Primers used in this study.

Target	Name	Sequence (5′–3′)^a^
ITS region	ITS4	TCCTCCGCTTATTGATATGC
	ITS5	GGAAGTAAAAGTCGTAACAAGG
GAPD promoter	GAPDpromoter-F	GAATTC GAATTCGAATTGGGTACTC
	GAPDpromoter-R	GGATCC GGATCCTTTGAAGATTGGG
eGFP ORF	EGFP-F	GGATCC ATGGTGAGCAAGGGCG
	EGFP-R	GCATGC TTACTTGTACAGCTCGTC
TrpC terminator	Ncterm-F	GCATGC ATCATTCCACTCAACATTCAGGC
	Ncterm-R	AAGCTT ATCATCATGCAACATGCATGTACTG
hph gene	HYG_5F	GGCTTGGCTGGAGCTAGTGGAGG
	HYG_3R	CTCCGGAGCTGACATCGACACCAAC
TAIL-PCR	RB1	GGCACTGGCCGTCGTTTTACAAC
	RB2-1	CTGGCGTAATAGCGAAGAGG
	RB3	CCCTTCCCAACAGTTGCGCA
	AD1	AGWGNAGWANCAWAGG
	AD2	WAGTGNAGWANCANAGA
	AD3	WAGTGNAGWANCANGTT
	AD4	WAGTGNAGWANCANGAA
	AD6	WGTGNAGWANCANAGA

^a^ Underlined sequences correspond to restriction enzyme sites introduced for cloning purpose: *Eco*RI (GAATTC), *Bam*HI (GGATCC), *Sph*I (GCATGC), and *Hin*dIII (AAGCTT).

### Plasmid Construction

Fluorescent protein constructs were made by using *eGFP*, enhanced green fluorescent protein gene. The primers used to amplify the promoter, *eGFP* coding sequences and terminator are shown in Table 2. Each primer contains a restriction enzyme site at the 5′ end to facilitate subsequent construction. PCR amplifications were performed using the FailSafe PCR system (Epicentre, Madison, WI, USA). The PCR products were isolated from gels, purified by QIA quick columns (Qiagen) and then cloned in pGEM-T Easy (Promega Corp.). All clones were verified by sequencing. *BamH*I and *Sph*I fragment of *eGFP* coding sequence (720 bp) and a *Sph*I and *Hind*III fragment of terminator from *Neurospora crassa trp*C (260 bp) under the control of the *EcoR*I-*BamH*I fragment of *Cochlioborus heterostrophus* GAPD promoter (466bp) was ligated into *EcoR*I and *Hind*III site from the multi-cloning site of pGEM-3ZF (Promega Corp.). Subsequently, the *EcoR*I and *Hind*III fragments were cloned between the *EcoR*I and *Hind*III sites of binary vector pBHt2 [8], named pSK1044 (Fig. 2 and Information S1).

### ATMT of *U. muehlenbergii*

Initially, *A. tumefaciens* strain AGL-1 was transformed with binary vector pSK1044 (Figure 2). ATMT was carried out as previously described [57] with a few minor modifications. Cells of *A. tumefaciens* were grown at 28°C for 2 days in 1 ml minimal medium (MM) [62] supplemented with kanamycin (50 µg/ml). After transferring 100 µl bacterial cells into 900 µl of induction medium (IM) [4] containing 200 µM acetosyringone (AS), they were grown for 6 hrs at 28°C. For co-cultivation, freshly harvested cells of *U. muehlenbergii* were used. After mixing equal volumes of *U. muehlenbergii* (1×10^7^ conidia/ml) and *A. tumefaciens* cells, 200 µl of the mix was spread on sterilized 0.45 µm pore cellulose membrane (cellulose nitrate, 47 mm diameter, Whatman Ltd, Maidstone, UK) overlaid on co-cultivation medium amended with 200 µM AS. Following co-cultivation at 23°C for 24 hrs or 36 hrs, the membranes were transferred to PDA amended with 20 µg/ml hygromycin B and 250 µg/ml cefatoxim to select transformants. Approximately one month later, individual colonies were transferred to 24-well plates (SPL, Korea) containing PDA. For isolation of pure culture, method for isolation of bacterial colonies was applied. All transformants were cultured in PDB, mixed with glycerol to make a 15% solution, and stored at –80°C for long-term storage. Colony color, size, and morphology of individual transformants were examined by culturing them on PDA.

### Genomic DNA Isolation and Southern Analysis

Transformants and the wild-type strain were grown in 30 ml of PD broth at 15°C in an orbital shaker (200 rpm) for 7 days. Genomic DNA was extracted using DNAeasy mini kit. One microgram of DNA from each strain was digested with *Hin*dIII, a restriction enzyme that can be used to determine single copy integration of T-DNA, and fractionated in 0.7% agarose gels at 40 V for 6 hrs in 0.5% Tris-Acetate-EDTA buffer. Fractionated DNAs were transferred to Hybond N+ membrane (Amersham International, Little Chalfont, England) using 10× SSC (1× SSC is 0.15 M NaCl plus 0.015 M sodium citrate).

Hybridization of Southern blots was conducted using the hygromycin B resistance gene as a probe. The probe was prepared by PCR amplification of a 1.4 kb fragment from pBHt2 [8] using a primer pair, HYG_5F and HYG_3R (Table 2). The resulting PCR product was isolated from a gel using QIAquick spin columns (Qiagen, Valencia, CA, U.S.A.) to produce a probe, which was labeled with ^32^P-dCTP by random priming (Rediprime II DNA labeling system, GE Heathcare Life Sciences). Hybridization was performed at 65°C overnight in 6× SSPE (1× SSPE: 0.18 M NaCl, 1 mM EDTA, and 10 mM sodium phosphate (pH 7.4)) containing 1% sodium dodecyl sulfate (SDS) and 100 µg of denatured salmon sperm DNA per ml. After hybridization, blots were washed twice in 2× SSPE and 0.1% SDS for 5 min at 65°C. Signals were detected by autoradiography using BAS-MS imaging plate (Fuji Film).

### Microscopy

To screen for expression of eGFP in transformants, conidial suspensions from randomly chosen transformants were dropped onto a slide glass and observed using a Zeiss Axio Imager A1 fluorescence microscope (Carl Zeiss, Oberkochen, Germany). A filter set with excitation at 470/40 nm and emission at 525/50 nm was used.

Scanning electron microscope observations of *U. muehlenbergii* hyphae and yeast-like cells (Figure 1) were carried out by fixing cultures with 2.5% glutaraldehyde in 0.1 M sodium phosphate buffer (PB, pH7.2) for overnight at 4°C and then treating them with 1% OsO4 in PB for 1 hr at 4°C. The fixed specimens were dehydrated in an ascending series of ethyl alcohol and subsequently sputter-coated with OsO4. The specimens were imaged using a field emission scanning electron microscope (FE-SEM; S-4800; Hitachi High-Technologies, Tokyo, Japan) operating at 15–20 kV.

### TAIL-PCR and Sequencing

TAIL-PCR was performed to isolate genomic DNA sequences flanking inserted T-DNA as described previously [52]. The secondary or tertiary PCR products were treated with using ExoSAP-IT® (USB, Cleveland, OH, USA) according to the manufacturer’s instruction and sequenced using primer RB3. Resulting sequences were analyzed through NCBI (http://www.ncbi.nlm.nih.gov/) to search for protein homology at the insertion site of T-DNA in each transformant.

### Mitotic Stability of Transformants

To determine the mitotic stability of transformants, 24 randomly selected transformants were cultured on PDA without hygromycin B. Following five successive transfers, colonies were tested for growth on fresh PDA amended with hygromycin B (20 µg/ml).

## Supporting Information

Information S1 **pSK1044.**
(PDF)

Information S2 **Fifty full genome sequences with RB-border sequences are generated by TAIL-PCR.**
(PDF)
