# Supplementary material for: Agrobacterium tumefaciens-Mediated Transformation of the Lichen Fungus, Umbilicaria muehlenbergii
Source: PLoS One. 2013 Dec 30;8(12):e83896. doi: 10.1371/journal.pone.0083896 (PMC3875497; doi:10.1371/journal.pone.0083896)
Supplement: Information S2 — Fifty full genome sequences with RB-border sequences are generated by TAIL-PCR. (PDF) [file pone.0083896.s002.pdf]

Information S2. Fifty full genome sequences with RB-border sequences are generated by TAIL-PCR

CCGCCTTCAGTTTAAACTATCAGTGTTTGA: RB-border sequence

>UmT-001

TTAAGGTTGCAGCTAGAGCAGCTTGAGCTTGGATCAGATTGTCGTTTCCCGCCTTCAGTTTAAACTATCAGTGTTTGACA
CCACCATAGAGGCCGAGGTTTAGATCATCAGATCCAAGTATTATTATTCGGCCTGTCATGCAGCCTGTAGCCTGAGCTGA
GCCTATTCTACAAGCGCGCATCAACAAACACTCATCTCTGCTTCTCCACTAAGATGAAGCTCTAGCGGATCCGATCCTGG
GAGGGACTTCTGAGGCGGCGATCAAAAATGGCAGCGCCGCTTTGCTATCGCCTGCAAAAAACTCCCGGCCAGCTTCACCT
CCCCAATAGCTTCATCATCCCGACAACTCAGACAATGATGTAAACAAGTCTCCTAACAAGAATCTCTTGTTTAGCATATG
TATCTTTGATGTCATCGCGTTGCTGATGACGGGAGGAAAGGGATGGTGGGTTGTTTGGACTCAGGGAAGCGTCTGCATTC
CCCAAGTCCCTCTGAAAGTAGGGACCAAGCATCTGATCCTGACTCATTCTGCTCAATCATTCACCTTTCCTGCTGCACAG
CTGTTGGGGAGGGATCCT

>UmT-002

AAGGATGCAGCTAGAGCAGCTTGAGCTTGGATCAGATTGTCGTTTCCCGCCTTCAGTTTAAACTATCAGTGTTTGACACC
ACCATAGCGCCGAGGTTTAGATCAGCAGATCCAAGTATTATTATTCGGCCTGTCATGCAGCCTGTAGCCTGAGCTGAGCC
TATTCTACAAGCGCGCATCAACAAACACTCATCTCTGCTACTCCACTAACACTGGCCGTCGTTTTACAACTGGCCGACTG
TTAAAACCCTGGTGATTGCCAAAACCCTGGCGTTGCCCAACTTAATCCCCTTGCCCCCCGTCCCCCTTTCGCCAACGGGC
GCAACAGCCAAGAGGCCCGCACCGATCGCCCTTCCAAAGAGGTGCGCAGCCTGAATGGCAA

>UmT-003

GAAACGGGATTGGCAGCTAGAGCAGCTTGAGCTTGGATCAGATTGTCGTTTCCCGCCTTCAGTTTAAACTATCAGTGTTT
GACACCACCATAGCGCCGAGGTTTAGATCAGCAGATCCAAGTATTATTATTCGGCCTGTCATGCAGCCTGTAGCCTGAGC
TGAGCCTATTCTACAAGCGCGCATCAACAAACACTCATCTCTGCTTCTCCACTAACACTGGTCGTCGTTTTGGCAGTGGG
CGACGTTTTAGAACCTGGTGACTGGTAAAACCCTGGCGTTACCCAACTTAATCGCCTTGCCCCACATCCCCCTTTCGGCA
GCTGGCCTAATATCCAAGAGGCCCGCACCGATCGCCCATCCCAACAGTTGCGCACCCTGAATGGCGAAGGGTAGACTGAA
TGGAG

>UmT-004

ATCTCCCGCTACGCAGTCTTTATATGACACTGACTTGGACAATTGTCGTTTCCCGCCTTCAGTTTAAACTATCAGTGTTT
GAAAACGAGCCCGAGGTCTAAAAATGATACTAGACTAAAAGAAGGGGGACGGTCGGGTGAGGACTGGAATGCTCCGAACC
TTTAGGAGCTCGTGATTGCATGGAGATAAGTGTTTTCTCTCAAAAAAGGTCTTGGTGAATGGTTTGCGATGAGGAGCAGG
GAACAGGAAAATGAAGGCTATTTAAAAAGAGTGGTTACGGATTTGATTCTCTCTAAACCCATCCCCCCCTGCTTGGTTTC
TCGGCGATCATCGTCTGTACTGCTCTT

>UmT-005

TACGATGGCAGCTAGAGCAGCTTGAGCTTGGATCAGATTGTCGTTTCCCGCCTTCAGTTTAAACTATCAGTGTTTGAACT
TCCGTGTACGATCCCGCAAGTTGTGCTGTGGTGGCTGTGATCCATGTCCTTATAATCATGGGGATATAGAGGGGTAAGGT
GCTAGATGACTTAATGAATCAGCTTATGAATAGTATATCAGTCAGTTATAACTCAGCAGTCTCTGCTTCTCCACTAAAAT
GGATGTCATTATTGAAAGAAAACTGTAAGTGTACTACTTGAAAAAAGCAGACCCTATGAAAGTTTGAAATAGCTTTATTG
GAAGGTTCACTTATTCTGTTAAACAAAGGTTCCTTCCATCCTGCACTCATTTGTTGAATTTATTCAACGGATTGACCAGA
GTTCGATTGCTACAAAAAGTGGTGCGAATATATCGAGGGGGGATGCCAAGCCATTAAGCAGCGACAGTGAAAATTCAAGA
CCATTTCTTCAAGCAAATTATCGCGTTGAAAAGGTAAAGTGTTATAAGCTGGCGCTGAAGGTCTATGAAGCTGACAGACG
CTCGGTGGAATGTATTTGGCTCTTAGCTTCGGAAGTAATG

>UmT-008

TCCCCTGGATAAAAGACTTGATGAGATAGAGCAGCTTGAGCTTGGATCAGATTGTCGTTTCCTCCGCCTTCAGTTTAAA
CTATCAGTGTTTGAAGAGGCGGAATGGGTGACCCGGAGAGGACGGAAGACCCTGAGCTGGAATCTTGGCGTAGGGCTTGT
AATCGATCATGATAGCCTAGATGCTGCCCGGGAGCAGTGGTTGGAGGCCGCATGCCCATGTGCTCTGGGTTCGACACCGT
CCGTCTCCTCTTGCGGGGCTTGACTGGCTCGTATTCGCACTTTAAGCCGCGCTCCTGACAGCGATCGCACTGAGGCCGCT
CTTCGTTGCATTTCTTCTTCTTTTCCTTGCTGCAGATTTGTCAGCTGTGACGTTCATTTTCCAACACCTGCCTTGGAGAA
AGGCTTACCAATGAATGCAGCCTTAAGGTCAAGCAAAAGAACACCGTCAGCTTGAATTCGCTTACTCTTCAACATATTGA
GGTTGAAAAATATTATAGAGACTTACCGGCTCTTGACCGGCGCTTAGGGATTTTGGCGGGTACGGGTCTCTGATACTCCA
CTAATGTGACTTGAGCGCGAGATACGAAATAGGGTTGGTACAACTCGACGAAGACGGTAGTATAAGAATAGATGTTTTCA
GAAAGAGCGGGAGAACAAGTAGGAGAGTCAGGTACTTTTTTAAGACGAGATAGAGGAGCAATAATACGGGTCCTTTGGAT
GCCATAGCTCGAGTAATATACTGGTTGATAGCAAGGCATCAATGGCCA

>UmT-009

ATCCGGCTATTGCAGCTAGAGCAGCTTGAGCTTGGATCAGATTGTCGTTTCCCGCCTTCAGTTTAAACTATCAGTGTTTG
ATCATTGCCGGCACTACCATGTTAGCTGAGTACGTGTTATTTGCATCATATTGTTGAGGGTTCAGGTTCAGTTCAATTGA
TTAAACTCATGTGTCTTGAGCTGAGAATGATGTGTGCATTACAAGTGATAATTAACTCAGCTGATACCTCCTCCTGACAA
AACTTGTGACTTGTACTGTGTAAGACTATAGCGTCCCTTACTAGAAAGGGAAGTATAGTCAAGGTTCATGACAGCATGAG
ATATAAATGTATAGAGGTTATATGATCCTAACTATATAGAACTAATAACTAAGGAATGAAGGTGAGATTGAGCAGTGCAG
AGTTTTCATGTGACTCACTAATGCTGTCAAGCCGCTTGTAGGGTGCATGCCTTACATGAGTCTCAGATTGTCATTAACCT
GTGACAATTGCCTCCCCTCTATGCAGCAGCCCTTCAGCGTAGAAAGGTGAGTGACTGATAAAAGACTCAGTTAACAATTG
AATTAATGATGCAACATCAACACTGACTAAGTGAGATGAAAGTGAGGTGAGCTTGTCACGTGACAGTGAAAACCCTGAGG
AGCGAAGAGGCAGCAGTGAAGTCATGACAGCATCAGCATGACAGCATTATGATAGCATAATCAAGGCCTCGAGTGATGAT
ATCACAATAAGAGCTTCAGAATATAAATACTGTCAACTCCTCAGCCTTCTGTCATCAACTTTTGCCAGAGCAGTGATTCA
TCATCACAACATCA

>UmT-010

GGATTGGCAGGCTAGAGCAGCTTGAGCTTGGATCAGATTGTCGTTTCCCGCCTTCAGTTTAAACTATCAGTGTTTGACGT
ACCCAAGCAGGCTTAGGCTGAGGAGGCTTGACTTAGCTTGGTGCCAAGTTTTCAATGAGCCGAGCCGAAGGCTGAGGCCC
CGCCAGCTGCACTCGGCTCATATCGTGATCGTGGACTTGGCCATTACCTTACCATAGTGTGTAGGCAAGCGCAATTGTTG
TATAATGCGATCATAACTCAACTTTCGTCTGAAATGGCCGAAGTCGTTGGTTCAGTGTCTGCAGTGGTGTCTCTCGTCGA
ATTCAGCGGAAAGATCCTGTCTCTTGGCTATGGGTACCTTTCTAAAGTTTCTAGGGCGCCTGCTGAAGTGCGATTATTGT
TGAGCGAGGTCGCAAATCTTAACGTCTTACTTGGTCGGCTGCAGGCCTTTGCGGAAGATGCATTTGATGCCAGCGCGAGC
ACTGTCCTTCAGTCATTGGCTCAACTTGGGATTTTCAAGTCTTGTGAGAAATTGTTAGGGTCTGTGTATGCCATCATTGA
TACTTGTCAGCGCTTGGATGGCCAAGATATGAAGAACTTCGGGAAAAGGCTTATCTGGCCTTTTAAAGAACGGGAGACGA
AGGATACTATGCAGCAACTAGGCCGACTTCGAGATACTCTTACCGCCGCTCTTGCTGCTGATTCTGCGTATGGCTTCTTG
GCTACACAGGGGTGTTAATGACTTATGGGGCCGCTAACGATATTACAGTGCCTCTCTGTTTTCTTCAACTAAGATA

>UmT-011

AGCAGCTTGAGCTTGGATCAGATTGTCGTTTCCCGCCTTCAGTTTAAACTATCAGTGTTTGCGAGCTGACGATGCCTCGT
TCCTGTTTGTGTAGCTTTGCGGAGTGGGAAACCCCCAACCACAAGCAGCGTCGATCTGATGGGTGCGCCTAGACCCTCTG
TTTGGACCTCTGGACATGCGTAAGTCTCAGACTGTTGGGCACGGTTTTTGCTCGTTGCTGTGCGAAGTACGGATACTGCG
ATACGCAACATGCGAAATAGATATCTACATCCTGCCGGTACTCCAAATAAGTATAAGCTCGACAAGGGTAGCGGAATGTT
TGCAAGCCCTGCTGTGCGATGAGAGGAGCTTCATTGCGAAGCAATTTTTAGTGTGGGAAAGGCTATGGTAGTCATCAAAA
AGTCAACAACACTGACAGATTAACATGCAGAGAGCAGTATCTTTTGAACATGTTTATGGTCTCTCCAGGGAACAGCCTGA
CCAGAGAACAGGTCGTCGCTTGGTTATGCTGCTATTGAGAAAAGCTTCTGCACGCTCATTATGAATTCTCCACTTGCTCT
GGATGGCCCACATCACATGTCCTGAAGCCAGCCGCGAAGGACACGTCGAGGAATACCCGGGAACCCAACCAACATCATGC
ATAACAAATTGCGGTCGGGTGGATTGGTTGCAAAGTACCCAGCCGTGCCTTTCCCACAC

>UmT-012

CCCGTGAAAGGTAGGTTACGATGCAGGCTGAGCAGCTTGAGCTTGGATCAGATTGTCGTTTCCCGCCTTCAGTTTAAACT
ATCAGTGTTTGATGAACCCTGCCGAGCTCAGGCATGTTGCTGTAACGCCCTCTCTGCCCCTAAAATAATAAATTGCGACA
TACCATTTGAAGAGGAAGCTTCCCATCTTGTTCTCTGTCGCAGACCCGGGCGGATCTGACTTCAGTATGGCAGCGATCGG
GTCGATATCAGAGCTTGAGTCTTCAGGTGTCTGCGGGTATCCAAGCGCGAACTGGTAGAGTTGCCTGTTGATGTTGCTCC
TCCGACTCACAGGTCGCGCATCAGACGGAGCAGGGAGGTTTGCGCTCGATTGCTCAGTCCCTGATGTGACAGGAGAGACA
GGCAACGAGGAACTTGATGGATCAATGGTGGCGACTACAAGGAATCACACTCGGCTTGAGGATGGGAGGTCGGTGGGAAA
AACGCGTGTCCTTTAATCCTGAAGGGGCGTGCGAATGAATCGTCTGAACAGTGGCACATGGAAAATGACTTTATTCAGGC
CAACGCTTGGCACCGCATCAAACGAGATTCCACAGATCGGCAGCGTCCGAGGGCAGTTCAGACGAATGAGAACCTGGTTC
CTGCTTCTCCACTTACACA

>UmT-013

ACGATGCAGGCTAGAGCAGCTTGAGCTTGGATCAGATTGTCGTTTCCCGCCTTCAGTTTAAACTATCAGTGTTTGAAATA
AGATATTCAGAGGTGTTTTGAGTTAGGCTTGAACTGACATGGAGATCCACAAAGATTGGAATCGCTGGTGATGTGGAGAT
GCCCTCGCCCGCTTGAAGTCCAAGGTCGGAAAAAAGATGGGAAGGTCTCAGGATTAGCACATCGATATGCGAAGCAACAA
TATATCAATACAGTTCTGAAAAGAGCAAAAATCGACTCGGACGGGACTCGAACCCGCAATCTTTGCCAATCACGTGACCG
GAAAGCAACGCCTTACCATTAGGCCACCAAGCCTATAAGCGATTCTTAAAAATTGCTTATTTATATCGATAAAGCTAAAT
GTTAAATATGGCTTGCTTTCGTCAGTATTCGGTAATCTCAAGAAGAACATGTTGAGATGCTAACAACTTTAGTCACTGAA
GGTCCTGAGCAACTCATGCTGTTGTAGAGTGTTGTTCAAGATTGCCAAATCTGACTAAATTTCAAGTGGTCAAGGAGTGG
TTCTGGCCTTAAGCTGCTATATAGTACACATGATAAGAAGCAGAATATCGCATTGGACGACGGTCTGGCGACGCCGAGGA
GCCTCCAACACAACCCACACATGAATAGGAGCGTGGCCAAGTGTGCAAACTTATATACAAGAATGAAAAGTGGAACGCGA
TGGATAGAAAGGACGCAAGAGCCGGTCTTGCGTAGGCCGCAGAAATTGAGAATATAGAC

>UmT-014

GACGGTAGGCAGCTAGAGCAGCTTGAGCTTGGATCAGATTGTCGTTTCCCGCCTTCAGTTTAAACTATCAGTGTTTGAAC
TGACCTATGATAGCACTGATCTCTGCTTCTCCACTAAGCCATTGCTCTGACCAGTCACTGAGGCCTCCTAACACCGTCGG
CCTCACTCAATACCCGCGAATGTGAAATTCCATGCGTTACCGATCCAGCGGCGGCACTTGCTATACATGCCGATGGAGCT
CTTACCCACTTTTCAGCTGTCGGTTGTTTGCGTCTCATGTCTCCATCAAAGTTTTAGTTTCAAAAGATCTCGTGCCTCTT
GTCGAGCTTCGCCCCCCAAAGAGTGAGCGGGCCGTGGGCCGGTCACAAGGAGCTGTTTGTAAATATACAGCCCAAAGGTT
ACTTCAGATGCGAATGGCGTTGAGGCAGTGTCGATGGAGATGCATTAAAAATAAAGTACAAGAATAATAATGGCTGTGAT
ACTGGTCACTGTGTTATGCACCGTTAGTGGTCCCGCTGGTTGCACAGAACCCGTTGAATAGGGCGTGCCCCATTCTCTAA
TGGGCGCCGCTTCTTCTCCATCACAATGAGTACTATGGATCACATCACATGGAGAAGCCATCAAGATGGGTGGCACGTTA
CTTTTAATCGCTAGCGATTCTGTTGCTTAACGTGGCGTCATACAAAGCGTGCCTAAAGGACTCTCCTCATGATCATGTCG
TCGTAATAAATTCCATCACTCTAACTCCAGGCCCGCATGCGTCACAGCTGACCACGGATCACCCCATCCTACAG

>UmT-016

ACGATGCAGTCTAGAGCAGCTTGAGCTTGGATCAGATTGTCGTTTCCCGCCTTCAGTTTAAACTATCAGTGTTTGACCCC
AGCCCTGCTTGATCACCGCCCACGGTGATGTCAAAGCAAATGCAATGGGTAGAGAGATAGTGCTAGTAGTGTAGGGCCCG
ATAACAGTGTAGTGAGCAAGGGGATTGACCCATTCCTTCTTTCCTCCGCATTGTAATGATGTATGCCTTTTTCAAAGGTC
TTGATCCAATACACAATCCTCAGTCCTTCGATTCGTTGTGTCATCTGCTCTCTCGGTCCTTCGATACCAGCAGGATTGCC
TCAGTCCATCGAGGTCTTATTCGCAACGCTCAGTCCTTCGAGTCGTATGCTGGATTCGGTCCTTTGAACCCGTCCCTCAG
TCCTTCGAGGTCTAATTGATAAGGTATGTCTCGGTCCTTTGAGATAGGTGGGAGGAATGGTTGAATTCGTCCAATTGATG
AGGTGATTGAAGATGACTGGCCTGGGCTGCCCCTGAATGACACAGAGACAGCCCCGCCTTCGGCTCCAATGAAAGGGTGG
TTTCAGGCAATAATGGTTGAACAGCTTGATTGCTTGGGTATGCTCGAGGTGTTGTTCTAACGAGGAGAAATTCTAGCTAA
TAAGCAGGTGGCTGACTAATCCCTCCACAGCAGCGACGGACGAGCTGGACCCTTGCGACCTTGCGACCTTGCGACCTTGC
GACCTTGCGACCTTGCGACGGGGATCGGCTACGGCAAGGCTGAGTAACCGCTATCAGGACTTGTA

>UmT-017

GCCAATGCAGACTAGAGCAGCTTGAGCTTGGATCAGATTGTCGTTTCCCGCCTTCAGTTTAAACTATCAGTGTTTGAGTG
AGCGCGAAGCTTTTCCTAGTCAGCAGTGCGCAGGCTTGGTAAGGTGGGCTAGAGGCTGGAGGCCATCTTAGAGGCCGGCA
GTGGGATTGGGAAGCACGCGGAACGTGGGGCTGGCCATGACGTTGTGGGCATGGTTACGTAAGCAGGGCGTTTATTAGTC

GAAACTTCGTAGCAGGCTTCATACCACCTTCATGCACAATACGATTCTATTGCCAGCAATTGTTTACCCACCAAAGGACC
ACAACGTGAAGCGGTCGGCCTAACTGGTAAGATACCGGTAAGGCTTGAGGAGGCTCCCGTGATTGGCCTGACGAGGCCTG
GCGGGGCGGGGCCTGGGCCATGCGCGGGGGCTCTTGTGGCCGCGACCCTGCATGCAGTCGAGCCAGTGAGGTGAGGTGAT
GATAAAGCCGATGGTGGACACTATCCCCGGCGCACACGTGAGCCTGTAACGACTTGGTAAGCAGGCAAGCACAATTGGAC
ACACGAACAACAGTTACGGCCCAATGGTTGATCATCTCGCAATCGCCTTCTATGGGACTTCAACGACTGCACCTTGTCTT
GCTTAACAACCCAACTCTGTTGCACAACCATCAAGTAGGTATTGCCAACTGAAGCTGACCCGAGCTGATATGAACGCATA
CGTGAAGACCGTTGTGAAGTAAATTGGACGGTATATAAGTAGAGCAAAAGCCTGCTCCATAATGTCTTATCATA

>UmT-018

TGGAAGGGAGCTAGAGCAGCTTGAGCTTGGATCAGATTGTCGTTTCCCGCCTTCAGTTTAAACTATCAGTGTTGAACGGG
CCTCTTCGCCAACGGCAAGACGAACATCAAATATCGAGATCGGGGACTGCTACTAGTAGCTGGCTTGAGGGCTCATCAAC
ACGACATGTAATTTGCTATGGGATGCACAATCATCAAAGCAGCCGTGTTACCTTTAGTTTGAGACTTCGGGTCCTTCGAT
GTGTCGTCGCCGCTCTCAGACTCCTCGTTCGCTCCTGCGGTTTTTTAGCGTCGCGTGGCCCGATTCCGCCCCTCACCCGC
TGACATTATCCAAGCTTCAGACTTCCCAGCTGTCTCTTCACTAAGGTACCTTCAGGTGCCGGTCACACTTACACCGAATA
CTCCTCTTGACCACCAGATTCCACGGTAACTTCCTGTGTGCATTTTACAGAGCTTCATCCGTCGCCATCCACTTCAGCGA
TCTGTGGAGAATCCCCAACCCTTCCCGAGTCAGATGGTCTACGACACTTGACACTCCGGCTTTCACCGCCATAGGCAGCT
GTACTGCAGATAGTCGCCTTCGGAAGATGAGTTCTTCCTCCAGTCTGAAGTGACTTGAGAGCAGTCTACCTCGATCCCCA
TGTAGCACCCTTGCCGTCCGGTAACGCGCTCCACCCTCGTGTCACCTTCGTGTCAACCCTTGGCCACGAAAGACTCCATT
TTCCATCAGAATGGATCCTCCACTGCGATCCCCGAGAGCAGCAGCGACCCATGTGTCACGCGTCGAGGTCACTCCGCA

>UmT-020

CTGATGGCAGATAGAGCAGCTTGAGCTTGGATCAGATTGTCGTTTCCCGCCTTCAGTTTAAACTATCAGTGTTTGATTGC
CCCGTGGGATACGTTACCGGTTTGAATCGCGATACCATTGGAATGGGGCGGTCTCTGACACTACTTTAATTTTTCGAACA
ACCTCGGTGTGATAACTGCTTGAGTGCATCAAGTCACTTTAATCCACCGATAAGCAAGAGTCATCCGAATCACACGCATG
ATACAAAGTTCTGAGTATCCTCCCTCTTTAGTGACAAGTCCTAGCAAGAATTAAACCATCACGATCGCCTTATGCAAACT
ATGGCCAGTCCTTCGACACACCCGAACCGAATATCCACTCAAATAGTAACACCAAAACCCCTGAAAGAACAAACGCGAAC
TATTGATACACAATCTCCCTGCACCGCCCTCCTCACAGTCTCCTCTCTCAGCACTGCTGCAAATAATTACTAGGCACCAA
TCCTGGCCTCCCCTTCTTCCCTGTAACTTCCGTAACCCACCAGCCATCATCCTGCACCCTCAGCACGGCCAGCACGTCTC
CCTTGGCGAAGCTCAGCTCCTCCGGAATAGCCGCATGATACATATACAACGCGCGAGCTAGAAGACATCCATCAATCAGC
CAAATCGCCTCCCAAGTTCAGGATCCAGCACTTACCGAAATGCAAAATCGGCCTCCCGTCTCTCGTGAACTGCATACCAT
CCGCAACACTCTTACTCCTCACCCTGACATCCGTCCTCCCATCCTGCCCCCCAGCTCCACCTCCACCGACCACCCC

>UmT-141

CATGTAAAGGGATTTAGAAGAGCTGAGCAGCTTGAGCTTGGATCAGATTGTCGTTTCCCGCCTTCAGTTTAAACTATCAG
TGTTTGAACGTTCAACTGCAGCGAATCATCTTCCGTCTCGCCGTCGACATGAATACGAATATCAACGGTGAAGTAACAGG
ACGAACAACCGTCTGTGAAGTACTACTTCATCCACTTTTCAAGATTAATAGAAAAATCAAAGGCGCAGCGCTGGTATCGT
GATCACTTTCCTCTTCCTTCCGTAAACAGCGAGTGCTCCCCTCTCTCTACTCCACTTAAGCAACGCCCCGCCTAAGCGTG
CAAAACGGCGCTGCATCGCGGCCTAACCCAGTCGATCGCCCTCGCCCTACAATAACTCCACATGGTTCGATTGACGGAAC
AATCCTGGCTCCCTCCTCCGTACTACCCACGAAAAAGCCGTAGTCTATAAAAACCTCCGTCTGCCACAAACCCCACCACA
TCCGGTCGCGATCGTCGTTCCCGGAGACCCTTTTACCTGGGCCGTGGCACTGGTTGCCTCCAATGGCAAACCGATAAGGG
GGCCTGCCACAATTTTTGTTGGTCACCAAACTCAACTGCGGCCATGCTGGCGTCATGTACGCTGGGATATTGAGACAAAC
GCTCGACTGACTATGGACTCTATGAAAAAGCACTGAACCTGAATCCATCGCCATCGCCATCGCCGCCGGTGTCAAGTGCC
GACCGGTGATTAGCGACCTGCGACTTTCAACTCCCCTAGTAAAGTGAACGGCCGGGAGCCTCTGGACGTGTTGGATGAGA
TGAAAAACAGTGAGAGGTGCCATGTCGGGAAAAATAACGTTGGATAGTTACGTGTGAAAATACGACGTCTGCCACGGCAT
GGCTCCATCGCGGAAGGTTCCATGTCATGAAGTCGCCCGTGAACATCGTTTGCGAGCTGATACCTTGAACTCTCTCGAGG
AAGATCGCCCCTTGATGTCTTCTGCTAGACACGCCTTGTCGATGCGAGGCAACTTCTTCTTCGTCCCCTACTTCCTCAAC
TTTCTGTTCCACTGGCGATTTGACACCGTGCGACAGTGCGTCTATCCTCGTCT

>UmT-142

TATATGAGTCAGATGAGCAGCTTGAGCTTGGATCAGATTGTCGTTTCCCGCCTTCAGTTTAAACTATCAGTGTTTGAATT

CCGTGAAGCGTCGAATCCCTGTGCGTATGGGCGTCCACATGAATACGAATATCTGCCTGCTTCACTTATTAAAAACTCCG
TCTGTGAATAACTACTTCATCCACTTTTCAAGATTAATAAAAAAATCAAAGGCGCAGCGCTGGTATCGTGATCACTTTCC
TCTTCCTTCCGTAAACAGCGAGTGCTCCCCTCTCTGCTTCTCCACTTAAGAAGTTCAAGATTGGTATCGAAGACTATCTA
CTCAGATATATAATGAAATCTTAGAGAATCCGCACTACAGCTTAGCATCGTTAACCTGTAGGAATAATAAAGGTAGGTGT
AGCAACGGGGGACACGCAGTAACGTTGATGAGATGAGGTAAGGGATAATTCATGGAAATGTTGTGTATAGAAGGCTTGGA
TGGTTGTAAGAAGAAAGAAATGTAGGGGAGGAGCAGGCAGTATTTGTAGGCGCGGATGATTCGTAAAACGCGGCCCAGAC
CTCCATTCACAGAGTGCCAATTCCGTACGTTTCCACCCCGGCCCACTTTTTCATCAATTCATCCAGTTGCTTTCATACTT
GACTAGTCTGCGAGGGGAGTGTTATAGGCGGCGTGAATATTTCAGATGGTCACGCCGAGGGCTTCACTGTAGGAGTGCGT
ATGTCTGGTGATCCTCTTAGCTGGAAGGGCACCCACTTGTGATCTTCTAGTCACGTATGAAGCACGAATATCGCACGTGA
ATAGCAGCAATGAATTGAGGTAGAAGGTAACGTAGAACAGCGTTGCATGTGACTAGGAGCGCAATGCGACTGTATAGTAC
ATCAGTAGCCATGTGTATGCCCTACCTTGCGTGATAGTTCGAGCCTGCAGAGACTGTCGCCATGTGATAAGGTCAACGCA
CACAAGATCACACTCTCTTTTTTGTTCCGCTGGAAACAGACCGTTGCTCTGAAACCTGCGACCTTGGGCCTGCGCTGCGT
TAAGACGACGGATATGACATCCTGCGTGGGCTTTCTTCGATATAGCATCTTATTTGTTATCCGCCAGACTCGAATGAAAG
TGTTTCGGAGCACTGATTAGGGAACGCAAAAAACAATCCTACTGTATGGCTTGTCAAGGCGACTTCGATCGATCTGCATT
CGGAACATCTACACTGATTCTTCTCGATTGCGAAACGTG

>UmT-143

GCTGTAAAAGGATATACGTTCCACTGACAGCTTGAGCTTGGATCAGATTGTCGTTTCCCGCCTTCAGTTTAAACTATCAG
TGTTTGAACGCCGTGATGACAGCGTTGATGAACCTTCCCGGTGCTGACGGCTTGCTTGATATACCTCGGACTACGTCGTA
AGTCCCAAAGGCTCACGGTGTGAAGGCTAAGGTGATTCTGCAAAGACTAAGACTGGGCTGGCAAAACTAGAGTGAGGATA
CAGATATCAATATTCAAGTAAAATACTCTCCCAATACATGTACGAGAACTATTCCTGCAGAACAGATACACAACCACCCC
TTCCACCACACCCCAGTCCCCCATCAATGTGCACGCCCCACCGCTTCTCCCCGTTCCACCAAGGCAGCGAGCTCCAACAT
TGCCCTCCCAGGGCCATGCGAGCACACAACCGCCTCTCGACACCAATTTTATTCTTCCAAACATTTTCTGATCACCCTCC
CCGGCGTGCGATCGAGACCAGAGTCCTAGGGACGTAAGACGTAGTGAACCATTCGAGCCTTTCCTCAGGTACTCATCCAC
TCGGCGGCCTTTCGGCCGAGCTGGAGGCATCCACTGGAAGGCTGAATTGCTTCGAGTCGGGTCTTTAGTATGTGGATCCC
ACGCGTCTGGTTGGTGGAGCGGGTGTGCTGGAGTGGGATGGTGTGTATAGTTCTCGATATGTATCTGTTTATGTATTGAT
GCGCTGCACCATACATGCGATTGTAGGATTCGATTCCGCCTTCGTACACGATTGCGCCAGCTCAGAACCAACTGGATATG
CTCCGAGACTGTGTCACGCTAGGCGTCGACCATGTAATCCAAGCCGTCGAATACCTCCATCATAAGCCCACATAACCAGC
CGTCCTCATCGCTTTCATCGCCTTCGCCGCCGAAGATTCTCTTCCGCTCCTCATCTAGCTTCTTTTCCTCTTCTTTCTGT
CGGTCTTTTCCAGAGAGCATATCCATGCGATGAACCCGGGTTGTGCCAGTGACGAGCTGTAAGATCGTTACTGAGCTGTC
GCGAGTTGGGCGGCCGAGAAGGGTAACATGTGCGATCTGAGATCAGACCGAATTGCGACTCAGTCGTAGGGAAATAGTAA
CGAGGGTTCGGTTGAGTATAGGCTTGACGAGCTTTGGTGCCGCCCGTTTACGTCGCATTGGTGGCGGCTCATGCTCACTT
AAGGCCGAATTAAAGTGATTGGAATAGTGAACTTTGTTAAAAGAACTGTGTGAGAAACCC

>UmT-146

GAAGTCAAAAGGATACAGCCAGATGAGCGCTTGAGCTTGGATCAGATTGTCGTTTCCCGCCTTCAGTTTAAACTATCAGT
GTTTGACTCGTTGCTCGTTGTCCGCTCGGTGTCCTTGGTGAGTGGTCCCCACCTCGGGCTTTGAAATTGACCTGTCAACT
GCCAATCATGCCTCGATGATGTCAAACTTAGAATCGCAGTAGCAGCAGGAGCAGCAGGAGTAAGAGCAAGAGAAACATGA
AAAAGGACCTGTGCGATGGCACGTTGTCACTGAGCACCTGCGAGACAAGCTTGGCGCTACCTCGTATGAGTGGGGCGAGG
CTCAGGGAGGTGTCTCTTTCTCTCCCTCTTCCCGTCTCTCTGCTTCTCCACTTATGGTCGGTAGCTATGGGCAGGGCTAT
GGCTGACACCTCATAAAAGCATGCTCGCTGTTGTGACACGAGGTTTCAGCGCCAGGTTCCATGGTTGGCTGATTCTCAGC
CTTTCAAGGCCTCGGGCCGACCGCCGGAATCGCGCCAGCGTTTGGGCAACCTTCGCAGGAAATGCCAATGATGCTGCAAT
CTGGTACCGCCACCACACGATCAGTCAGGCAGGATTGCACCTTGGCAACCCCAAAAGTATCGCGACTGGAAGGAGAGTCG
CTGAAATACTTGGGGTTTAGCAGTTGAATGCAGTGTAAGGTAGTGCTACGCACCTAGTGAGCCCGGAATGCCAGATCTTC
CGCTCCTAGCCCTGATCAGACAGAGCAAAACTCACTTTCACCTTCTACCCTATTTGGCATGGTGGTGGAGTGCTACATCC
GTTGAGTTGCGGGATGGCTTCATAACGGAGTCCCACTAATCCTCGATTATCGAATTAGCTCTACATAGCTCCGTGGTAGG
TGTACATGCGATCTATGTAGTACTAGCAGGACTGACTTCTCGTTGATTGACCCTATTGGTTGCTATTCTTCTTACAGCTA
GTACTACACTACCAAACAACTGCTAAACGCCTCACTGGCACAAAGAAAGCGATGACAGGGAGGAGACACAGCAAGCCCAG
CACGATCGAGAGAGGAGTGCCAGAGTGAGTCGAGATTGCTACTCATCGAGAATACACGGGGTGCGCGGGGATCATGAAGC
CCAGAGGCACGATGACCTGCATGACTGCATGACTGCATGCCTGGAACGGACGACGATCTGCAGCATACGGCATGCATTGT
GCGTCGATATTTCCCTCGGATCCAACACCGAAGC

>UmT-147

CCCTTGGAAAAGCTTACCAGCATCTGACAGCTTGAGCTTGGATCAGATTGTCGTTTCCCGCCTTCAGTTTAAACTATCAG
TGTTTGATGTTGATTTGCGGGGCAGTAGGAAGCTCGTGGACGGAGGGGCGCGAGGAGCGTCAATCATCGTGACAATTGCT
AATAAGGTGCCTCGGCGGTAATCCACCATGGAGTATGACCGCCGTAGAACATCTCCCACATCCTGTCTATCATCCAAATC
CCCACCGCCATCTTCACTTGACCTGTTCGATCTGCCATCTCTCTTTCTCCACAATGACGTGGTCGTCGTTTTAATGCTAC
TCGACTGATAAAACCCTGGGGTTGGCCAAATCCTGGGCGTTGCAAAACTTACCCCCTTTGACCACAGCCCCCTTTTCGAA
AACGGCCGTACCAATAAAAAGGCCCCACCGATGCGCCCTTCCAAAAGCGGGCCCACCCGGAGGGGGAAAGGCTAAAGGAC
TTGGGCTTGGATCCATATGTTTTTTACCGGATTGAATTTTAACGATTCGCGGTTTAAAGGAAATTCGTGGGCCGAAGGAC
CCGCGTGGCAACAGGCCCCTTGGCAATGCAAACAAGGTGCCTCTTCCAAAAAGGGTGCTGCGGTGGAACTCACCTAGAAA
ATGTACCAGCTGAAAAATTTTTCGAGATCCTGTCTACATCCTAAAGACATCGGGAACATCAACTAACACGGTTACACCTG
AATATCGGGTTGCCATAAAAAAGTCGGGGGCGAATAAAGCAGTTGGGGAATAGAGCCGGGAAGGTTGGCAACGGTAAGCT
TGTTATCGATACTCCTCTTCG

>UmT-148

GCGAAGGTAAAGCTGAGCAGCTTGAGCTTGGATCAGATTGTCGTTTCCCGCCTTCAGTTTAAACTATCAGTGTTTGATCT
AATTAATGTAGATCGCTCGCATCACTTTCCAAGTCGGGACTCAGATCTTCCTCTTCTCTTCATGATGGTAGTAATGATTC
TCGGCCATCACCCATGGAGACGTTGTTCTTCATTTATGGAATCCGGATCCCGGCGCGCAAGTCTGTCTGCCCGCCCTCTC
TTACTTGATGTCCTTTCTAGCCCGTGCCCTCTATGTTAAAAAATAACGATCTTCTCCGTCTCATGACCCGGAAACTCCAC
GGTGATTGGAACCTCCTGAAAGTGCTGGACTCTGAAACGAAACAGTATATTCGACAAGCTTCCTACGAGGCCAGAAGAAT
CCCGCGCGGGCCAAACAGGCTCCGCAAGATACACTGACTTTCTCAGGGGAACTACCATGGATTCCAAAGCTCAGCTCACG
ATTGGCGACAAGCAAGGCGCATTTGCGTAGTGTGCTCATGGTCGTCAGCGAAGCCTTTTCCGTGCGCAAGATTGAGGGCT
GGTATCAGCGTCAACGGTGATCATGATGGAGGTCCACAGCTTGGTGCGGCTACCGGAGCTGACAGGCACCATGCGCGAGC
TAAGTCAGGAGCTGGTGAAAGCTGGTATCATCGTTTACATGGCTGGGGATAGTTTACCAGATGATCAGCTAATGGAGGGC
GAGGATGAAGAGGCCGAGACGGAGGTGGATGATGTACTTGCCAAAATGCTGGTGTGAAGGTTGGGGACGTCCTGGAAGGT
GGACGACAAACGTCTGGACGAAGACCCGACCGAGATCTAAGTCGATCTAGAGGAACAAGATGCGACGTTGGCGCATATGA
TAGGCAAGCTGGAAGCGCTGAAGAGCTGAGCTGGGCCTGTTTTTCTGTTGTCATTTGAGTTCTAAAACATGCTCTTTGGT
GTTCCCGTGTGGGGGGGGGGGGGGGGGGAGTTTTTAGACTTTTTTACGAAATTTTTTCCCAAAGAACAAAGACAAGCCTTC
TACACCATTTAAAAACCGGGGTGCTTAGGTTGTCCGGAATCCCTGGGCCGCGTATGCCCGCCAGCCAAAATACGTATTGA
AGCTGCCGAACGAATCACGTAGTCACTTCTATCTCAGTCTATGAATATCTTAGAATGACTTTCGCTTCTTGGAAATTATT
CTGGAAACGGAGTCATTCGACAGTGAGAGTGTGTTCCTACCAACACAAGGAGAACTGTTAATTCCTTGGAAATCCTTATC
CCCAAGGCCAACCCG

>UmT-151

GATGTCAAGGACTAGGACAAAGATGACAGCTTGAGCTTGGATCAGATTGTCGTTTCCCGCCTTCAGTTTAAACTATCAGT
GTTTGACGTGTGTTTTCCGCGCCGACGAAGATCGTGAACTCCACGGGCGTGTCGACCGTTCATCATTAAGACAATTGCTA
ATAAGGTGCCTCGGCGGTAATCCGCCATGGATTATGACCGCCGTAGAGCATCTCCCACATCCTGTCTATCATCCAAATCC
CCACCGCCATCTTCCCTTGACCTGTTCGATCTGCCATCTCTGCTTCTCCACTTAGGGATCGGATGCGGATGGCCCGACAT
CCTGCTCAACCGGGCGAAAACACCCCTGTGCTGGTTCCCCCCGATTCGAAGCCGATAACTTTGTCAATGTGGGCGGTGCC
GACTAGGCTTGGAACGTTCACCGACCGAACACCTCGAGCTATGTATGCCTGGGGTCTCTGCTTCTCCTCTAACTGGCCAG
AGAGAGGATGGAGACTACGAAACTATGGCAGATTATACGGAAGATGCCCAAGGGAGCTTTACTACATGCCCATATGGATG
CTATGATTGACGTGCAGTGGCTAATCGATGAACCACTGGGCACTGGCTGCATGTGTATGAAGGCAAACCAGGCGCTGCAT
AGCTCAGAAGCGCGGGACAAATTGGTCGTCTCGTTCAAATACTCCAAGTCGCTGCCATCGGGTGCTGCGTCGATATGGAG
TAAAGAGTACCAATCAGGGCAGCTTGTCTCCATGAAAGAGGCAGCAGAGAGCTTTCCGGAGCATGGACGTGCTGGGTTTA
AGGCTTGGCTCAGCGATAGGTGTACCATCACATCGCTGACTCACTTGAGCACCACCTTGTACTCCACGAGATATGGCAAA
GTTTGCACTATGCTTCCCTATATTGAAATCGCTGCATCTTTTACGACCAATCTTTCAGGCTTCGATAAGACATATGCTAA
AGATCTAATGGTCGACGGCGTACGGTGGGTAGATTTCAGGTTAGCATTCCATTTCTAGTGACAGAAAAGCCGGCTGCGAG
GACCCTGGAGATGGCTACGAGGAGATGGTTCGAGTCTTGGGCGAGGAGTCGAGAAGTTCAAAGGTCAGACGAACGGCAAT
GGTTCTCTGGCTTCCTTCACCTATACCTC

>UmT-152

CCTGGTCAAGGACTCATTAAGATGACGCTTGAGCTTGGATCAGATTGTCGTTTCCCGCCTTCAGTTTAAACTATCAGTGT
TTGATCCCGGACCATGTGATCGCGCTTCTCGTTGGGGTCTCTGCTTCTCCACTTACCCCCTTTAAGGTTTTCTCATCATT
TTTGTCGTACGTCGCAGAACAGTCTGCCCGTGGAGGAAGAGAGTTTTGTGGCTCATGCGGTCAAGGATCACATCTGACTT
CATTGGAGATCGCTCCCGATGCAAGAGAACGAGGGTGCAGCAAGCGCGGGGTGCAGATAAGAGAATGATCCTTACGTGGC
GAAGAACCTCTCGCTGGAAAAGTGCCATTATTTTTTCACCCTGCGCTAGAGATGTTGTTTGCCACCACGACGTCATGTGG
AACCTTTGGTGGATGGATGGTTTCATGATTGTAAGATTCGTGTACTTGATCTGACCTGTCAGATGAAGCGAGTGATATCC
AAGACGGTATCGAAAATGGCCATTTCCGTCCTTCTAAATGTGTATATAGACCAAGCCGATCAATCCTCCTTATGCCCTAG
AAGCCAATTGCCTTGCCTCCAAACCCCCAATGTGTATTACATTGAATCCAACATTGTCCCTTGCCCGCGCTTCCAACGTA
GCAAAAGTGCCCAATTCCCTAGATCCTGCGCTCTTGATTCCAATCGAATTTTGCTCCCATTCGAAGTCTCTACTTCTGCA
CTAACCAGCTCTTGCCATCATCTTAAGATTGAGAGCTCTTGTCCGTTCCTGAATAGAGCTTTTAGATAAAAAAAGGGATT
GAGCCTCTGGATTCGATTCTGTTTTTGATGATCTGTTTCCGGGCGCGCACCAAAACCGGCGATCTCCTGAACACTTGCTC
TAGCTCTTTGAAACACACCGATTTAACCGCAATCATCGAACTCAGGGATGATGATTGTTACAGAAGTTATGGCGTCTTAT
GCTGTGGGCCTCCACGCTGCTGTGTGGGGAGGGCAAGAGCAAACCCGGTCATTCAGCACGACCATGGCCAAGCTCATTGC
GGAACGGGGGGCAATAACGTAACTTTC

>UmT-153

TTGTAAAAGGATCAGTCAGCTGAGCAGCTTGAGCTTGGATCAGATTGTCGTTTCCCGCCTTCAGTTTAAACTATCAGTGT
TTGATCACTTCTAATGCTTTAACCTTGCCCGGGACCATTGTACTTCTGCCCTTTCTGGTTCTCTGCTAAATTGCTATGAT
GCATTAATAAATGATTATGGATGTAGGTGGTTTCCAATTAACATTTCTACACAGAAAATTTTGCTCGTGCGAAAGGCTGT
GATGGTGGACCTTCAATCCCTCTACCCCATGCTAATGATCATAAATCTATCTGGAACTTCCCCCCCTTCACCTATAAAAT
CTCCCCTGATTCGAGATTTCCCTCGTCTCCACCTGAAAACCCCCCTGTTGGAGTATGACTAGTATGGCATAATTTGCTGA
ACGGTGCTGCTCGGAAATGGTTGCCTGCATGGGCTATCTGCTGCCTACTTTCTATCCTGCTCCCCCTTCTCTGCGGCGTA
CACTAATCGCCCGCAGAAGCGCGGCCGTCTGGACCGATGGTTGTGTAGAAGTACTCGCCGATAGTGGAAACCGACGCCCC
AGAACTCGTCCGAGGGCAAAGGAATAGAGTATATGCCGACCGGAAACCAGTTTCGAGTTTCTCCATAATAATGAGTGAGT
AGTTCCCAGATAAGGGAATTAGGGTTCCTATAGGGTTTCGCTCATGTGTTGAGCATATAAGAAACCCTTAGTATGTATTT
GTATTTGTAAAATACTTCTATCAATAAAATTTCTAATTCCTAAAACCAAAATCCAGTACTAAAATCCAGATCCCCCGAAT
TAATTCGGCGTTAATTCAGTACATTAAAAACGTCCGCAATGTGTTATTAAGTTGTCTAAGCGTCAATTTGTTTACACCAC
ATTCCCAACTAAGCCCGTTTAGGCAGCTGGGCTGAGCACGGTCGCCATCCTCCTGCGCCTAAAATTAGGTGTGCGACGGT
GCAGCTCGTGCTTACACCCTACCGAGTCGGGCCTGGCTTGACGTCGACGTCTGACGGCTCCGAGACAGCCCGGGCAGTCT
AGCTGACGCGGCACCAAGCATCTTCTTGATCTTGTGCTTTACATTTTTTTCTAACACGTCATGATGTTCTGTCTCACCCT
CAGGTCTTTGTGCTATCCTCCACTA

>UmT-156

GCGCATGTAAGATGAGCAGCTTGAGCTTGGATCAGATTGTCGTTTCCCGCCTTCAGTTTAAACTATCAGTGTTTGAGTAA
CGGAGGCACGGAGGAAACCGTTTCCTATCAGTTTGGTGCTAAGCGAAGCAAAGCGAAGCTACGAGTACGTACTAGCCGTG
CGAGCTAGGTAGTAGTAGTAGGGATAGCTGCCCACCAGTGCCACCTTCAGGTGCTCTTGGCTACTTCGTGATACCAATAG
TAACGTCCATTACAGCTGTAGGCGGGCCTCCCGGGCCTCAGTGTCCGTCACTTCGACCACACCTGCCTCTCTTCCCACAC
CTATCGTACGCCAGCTCTACCCAAGGCGATATACGAGGCAAAACCTAATTGTCCGGCGCTGTGATCAGGGACCACCAGTC
GCTCTTTCCTAGCTCATCGGTGAAGATGCTGTTGGGGGAATGAACCCTCCTTCTTACTGAGAATATCTTGTTCCCCTCCA
CTTAATAGGGGCTCGAAGCCACGCCCATAAACGTAGTGTGCACCTGCTCGTCAGCGAAGCCTTTTCCGTGCGCAAGATTG
ATGGCAGGGTAACATCGGCAACGGTAATCATGATGGAGGTCCACAGCTTGGTGCGGCTACCGGAGCTGATTCGCACCATG
CGCGAGCTAACTCAGGAGCTGGTGAAAGCTGATATCATCGTATAGATGGCTGGGGATAGTTTACCAGATGATCAGCTAAT
GGAGGGCGAGGATGAGGAGGCCAACACTGAGGTGGATAATGCAGTTGCGGAAGTGCTGGCTGGGAAGGCTGCCGACGGTC
ACGACGGCTGACGACAAGCGTCTTGCACGAGACCCGACCGAGAACTAAGTCCATCTTTGGAGCATGACGCGACGTTCGCG
CATATGATGGGCAGGCTGGTAACGCTGAAAGCTGAGCTGGGACTGTTTTTTGGTTGTCTTTTGGAGTTACTTCAACATGC
TCTTTGCGTGTCGTGATTTTTTGTATTTTTTTTTT

>UMT-157

GACGATTTAAGATGAGCAGCTTGAGCTTGGATCAGATTGTCGTTTCCCGCCTTCAGTTTAAACTATCAGTGTTTGATTGT
CGGCTCGCAGGCCGGGTCCGGCAATGACGTTGCCAGTCTTCCTCGCTTCTGTCGCATACCGCTCCTTGCGGTTAGACTGG
CGTCGCTCAGTAGCGCCGTCTCTCCGTCTACCTTGGCGTGTTTCCGTCACGTAACTTGGAGCCTCGCTTAAACGCGTGGG

ACGTGAAGTGCCAAGGGGAAAGTGAGGCTTGGGAAGAAGGCATTCGGATCTGGTCCCACGGCACAAGTGTTGCACCTCTT
CCATGGCATCCTTGCCACCGTCGGACTTACCGTCTTCCTTCGATCCTGCCATATCTCTGTTTCTCCACTTACACCAGTCG
CTCTTTCCTAGCTCATCGGTGAAGATGCTGTTGGGGGAATGAACCCTCCTTCTTACTGAGAATATCTCTGCTTCTCCACT
TAACAAAGTGGGCATACAGCACCGGGCGTGAGCGGTGGCAGATGTGTTCAGGAGCGACAAGGACTACCAAGCTGGTTCGT
GTCCTATAGCTTCGTTCTCGGAAACATCAGGCCCAGCATACTGAGCTCCGTCTCTGCAGATCCCCCAATGTTTAGGCGCT
TCTTGCCCAACATGTACGAAATCTAATTAGAGACATACTTCGGACCTGCGATATCTGGAAACGAGGCACCGGGGATCTAA
ATAGCCGCTGGTCTCGGCTCGAGGAAGTCAAAGTTATAGGGGATATACCCGGATTATACATACAAAAGTAAGG

>UmT-158

GTGTAAAGGACTAGGCGCAATGACGCTTGAGCTTGGATCAGATTGTCGTTTCCCGCCTTCAGTTTAAACTATCAGTGTTT
GACGGTGCTTCATGCAACAGGACATGGTATAGATGATCTGGCCCATTGCGTGCTGCAGCTGTAGATTGCATTGAACCATC
GTGCGTGATCGTAATATCCATCGGGGATCCCCCGATCACGAGTCTGACTTCTGATAGGGTTATCGGTGCTGAGTTCTCAT
GGGCTCGAGAAGTGATAATCAACTGTGAAGGGAGATCCTCGCCCACATGTCCTTCTGCAACACCGAATACGAAAGTAACG
GTTACTGCCATTGGGTCAGACATAGAGACAACCGGCTCACAGCTCTCAACTCTAACTTACAGCATGATGCAATGTCTTCA
GCTTGTAACACCACGCTGGGGCGAGACTTCGATAGTTGCAGACCGTCCAAGCACGTTGAAAGGTCATACCGCCAGCCAGG
CCTAGGCTTTACAGCTGATCGGGACAATCGTTAGCAGTTCATCAACTTTGGGCGAAACCTCACACAAGGCATCCAAGATG
CAGTCCTCACCTCTGCTCATCAACTCACACTCAACAAAGAGCAATGTCTCGGCATCTCCGGTATGGCGTGCCGATTCTCT
CAACAGCCAAGCCACTTCTTCCACCAAGTGCCACCAACCTATCCTCCCCCACCTAATAAAGCCAGCTGCCTGGTGTGCTG
ATCCCGCTAAAATACAACAGGCCTATGCCGTGAGCTGAACTTTGTAGAAACGAGATGGCGAGGCTATATTCCCGCAAGGA
CATATATATGACGACGTAGGTCGGCAGCTGAAGGCACTAGATTTCTTAGATCGTCCTAGAGCTGAATCGGGACAGAGAAG
CTCTTAAACGTCTGAGACCAAAAAACTTCAAGACGGACGGCTGAGTTGAGTACAGCGTTGTCATATGCATGCTTGGGTAA
AAAGGAGAAGCTCCGGCTGCGCTCTGAAAAAAGTCACCGAAGGCGAAATTCAGCACGGCTGACATGTCGAACCCCTTACC
GAGCAATGACATTACCCAGTTATGGGCTCCCAGCGCAGATGATATTATGATCTCTTTCTTGCTCTGGCACGTCAAGATAC
TACATGGTAGCTCATGGAAGGCAAAATCACGGGAGGCTTGAACTCCTAGATAGTAT

>UmT-160

CTATTAAAGCTGAGCAGCTTGAGCTTGGATCAGATTGTCGTTTCCCGCCTTCAGTTTAAACTATCAGTGTTTGACTGACC
TTCCACGGCAACCTGCTACTCCACTAACACTGGCCGTCGTTTTGGCACTGGCCGTCATTTTACAACCTGCTACTCCACTA
ACACTGGCCGTCGTTTGGGCCCGGGCGGCCTTTTTCCAACAGGACCTCCCCTTAAGGGAATTGGAGCTTTGGACTGGGAC
TGGGTTTTTTGTGTTGTGTAGTGTTGTGTTGTGGAGTGTATATGGGGATGATTTGGCACTGGCGGAAATTTTTCAACCTG
GAAAATCCTAACCCCAACAACTTTAAAAACTTTGGGAATATGACTTTCAACTGGCCCCATCACGAGAAGTTCCTCGGGAT
TGATTACCCCTGGGTTTTTATGGGAGGATGGAACCCCTGCTGCTCCCCTAATGTTGATACGGCCTGGGTTTTGTGGGAGG
GGGAACTCCTCTTATTCCTAAAACAACATCTTTGAGAACGTTGGGGATATGACTTTCTCCTGGGCCCATCACTAAAAGTT
CCTCGTGATTGATTACGCCTTGGCTTTTATTGTACGATTGCATCTCTGCTTCTCCACTAAGAGGGATGAGCTTGCGCGAA
TAATGGAAGGGTTGAATCACTGATGCGCACGAAATACCCCTACCGGCTGAATGTTATTGCCGCTAACCACATTATATACC
CACCTAAAGGGCTGATTTTATTATATATAATATGTGCCAGGTAACCCGATAGAGATTTTAAAAAAAAGATGTAGATTGTA
GCCTCATGGCATTTCAATTCAATTTTTGATGAAATGTCTTACCGTGCGCTGACCCTAAAGGGCTAGCTCCCTGAACAGAT
GTTTAGTGCTGTGTGAAAGCACGCCTTTTAAACCACCAAGATCAATACATCAAAAACCTGATGGTTTTTCTACATTAATT
ATAACCTAATTCTGCTGTCTGCCTCC

>UmT-161

ACCAAGTTCGAGCTGAGCAGCTTGAGCTTGGATCAGATTGTCGTTTCCCGCCTTCAGTTTAAACTATCAGTGTTTGATGT
TGATTTGCGGGGCAGAGGAAGCTCGTGGACGGAGGGGCGCGAGGAGCGTCAATCATCGTGACAATTGCTAATAAGGTGCC
TCGGCGGTAATCCACCATGGAGTATGACCGCCGTAGAACATCTCCCACATCCTGTCTATCATCCAAATCCCCACCGCCAT
CTTCACTTGACCTGTTCGATCTGCCATCTCTGTTTCTCCACTTACTAACCCTCTGCGGATGCCTTAAGACCATCCTCTGA
TCGATCCGATCACCGCTTTCTATGCCTTCCTCTCTGCTTCTCCACTAAACCATGTTACAAAAGCCCAGGACCTCCCGATC
AAATGATCTCGCTGGGGTACCAAAGAGAAACTACTCACCAACAAGGGTTTAGTTTCTGCCATTCGATACCCCTGGCCACT
ATTCTCGCGAGCTCCTGGCTCTGCTACTCCACTAAGGAAACTTGGTGGCATATCGTCTACCAGCATCGCATGCGGCAAGA
ATAAATAAATCTTCGGATAACACTGCTGAACAGCTCCTTTCTCATATGTAACTTGAGAGCTGCTGACACTAAAGAAGACT
GACTCTCTTAGTTCCTATCAGCCAGTATCATATTCTGAACATATGAAATGTACTACCTAACTTCTGATTGTAACTAATAT
AATAGCAATATTCAGTATTATCATTATGATTCTCTGTCCCTGCAGCTTCAGAAAGATTTAAGAGAGTAGACTCGGTTGTG

ATTCTTCTTAACAGTAAATCATTAGACAAAC

>UmT-162

TCTGGTCAAAAGGCTCATTAAGCTGAGCAGCTTGAGCTTGGATCAGATTGTCGTTTCCCGCCTTCAGTTTAAACTATCAG
TGTTTGATAGAGTCAGAAAGATCTTCAAACATAAAGTATCTGACAAAACTGAGACTTAAGTGTCTGTATAAAGCAACTGA
AAACTTCTGACCTAACACTCTTTGAGAATAATGTCTCATCACAGAAGACAGAGCATCTGTACTTAATGTCTTTCTCTGAA
AAGAGAAGAGTAAAGTAGAAGGCTGCTGAGTTCTCAGATCATAATTAATCTCTAAAAAGTGCATAAAAGGTAGAACTAAT
GCAGTATAATATAAAGACAAATAAGAAACAGATTGAGAAAGATACTTAACAGCAGATCTTTCTTTAACAGTAATATGCAG
CTGCTTAGAATATTTCAGTTTAATCATAAAGAGATTAACTGTACTATCAAGTAGTAAATTTCTCTGATTTGCTGTCAGAG
AATTACAATATTTCAGAGTACTTAACTCTGTTACTCTTGAAGGTAAACCTGCTGTCATATGATATAACAGCATAAGAATC
TTCAAGAATGAATAAATCTTCTGATAATACTGCTTAACAGTTCTTTTCTTAAACTGTAACTGAGAGTTACTGTTAAGAAA
GAAGAAAGAATCTCTCAATTCAGCATCAGTCATTATTAACTTCTGTACAAAAGAAGTGAAGTGACTCAACTTCTGATTGT
AAGAAATATAATAAGAATAAGCAGTATTATCATTGAGATTCTCTGTACTTGCAGCTTCAGAAAGATTTAACAGAGTAAAC
TCAGATGTAATTCTCTTAAACAGCAAATCATTAAACAAAATACTGTGTGCATCAATAAGCAGTGTCTTAAAAAGCAACTG
CATATCATCAACAGACACAGTATACCGTTTTTCTCCCTCTCACTAAAAACC

>UmT-163

TCTTGTAAACGTCTCAGGAGAAACGCTTGAGCTTGGATCAGATTGTCGTTTCCCGCCTTCAGTTTAAACTATCAGTGTTT
GACTCTCTGTCTGCTTCTAACTAATCGCTAGGTAAGGAGGTAAGGTGGTAAGGTATAGGTAGTTGCAGTGTAATGTACCT
CTACGGAACTACTCCGTCCCGGCTCCTGTACGGTCGGTCTACGCGGTTGGGGAGAGCGGAGCATCGTGTGCAGCGTGTGC
AGCGAATGTGGGGCAGGTATTGTACGGGACACGCGTATGGATTTCCTGCTTCTCCACTAACACAGGCCGTATTTTTGGTA
CTTGATCGATCCGATCACCGCTTTCTATGCCTTCCTCTCTGCTTCTCCACTTACGGTACCGCTTTTAGGTGCCCTCTTAG
CCTGGTTGAGTTGCATTCCCCGACTAACACATACTAGAGAACCCTACCTATTCAGCTTTCTGGTTTCCGAGGGCGCACTT
TTGGGAAGTCATTTGCTTGCCCTGGGTCTTCAGTTACCCTGCGGCCTTATCCTCTTCCTCATTACCAATCAGCAAGAAAG
ATTAACTCGACCCCACTAGACAGCGTATCAGCTCGTTTCTTACCCTGTAGCTGATCGTTACCGGAACGAATCCGTGATTC
TCTCTTAATTCCGCCTCAGTCAGTATGAATTTCTGTACAACTGATTGGAAATGACTCATGTTCTGATTGCACCACCCATA
ATACGAAGACGCAGCATTATCATTGATATTCTCTGTACTTGCAGCTTCATAAAGATTTAACAGAGTAAACTCGGATGTAA
TTCTCTGAAACAGCAAATCATTACACAAAATACTGTGTGCATCAATAAGCAGTGTCTTAAAAAGCAACTGCATATCATCA
TAGACACCGTATACCGTCCCCCCCCAATAAATACAGA

>UmT-164

CCCCGTGGGAGTATAAGCAGCTTGAGCTTGGATCAGATTGTCGTTTCCCGCCTTCAGTTTAAACTATCAGTGTTTGAGCC
ATTTGAATGGGATTTTTGCTTTCATGGGCAAGCAGCCATGTTGTTCGCCAAACGCTGTCTCGACCTGCGCAATGAGAATT
AAGTTTAGGGCAAAACAGTCAAAACAGTCAAAAAGGATTAACGTACCCTATACTGAATCCGGTTTTGAACATATCGGTAG
AAGAAGTATGCCAGCACAAGTGCGAGCACTGTGAGTCGGACACTCATCGCACCCGCGCGGGGGGGGAGGTCCTTGAGGAAC
CTTAGCTGCAAGCGAGACGACAACGGAATTCGACATGGTCAAATTGTCGCCGGAAAATCCTCCGTCCTTCTCCACGGAAG
CCCACTGCGCAAATATACAAAGAACATATCAAAAGGGGGGTTGGACGACCAGTCTTCCAACATGTTCGGCAGTTAGACGA
CGCCGGTCTTCCCCTGTGCCGATGCCTCAGCCTTGATCACGACCTTGTCGTCTTTGGGGGCCAGATTCAGCCGGCCACGT
GGGCCTGCGCATCTGGCCAATCGCACGGCAGCACGATGACTGCACAGCTGCCCCTCAACGCCAATAATTCGTCATCCTCG
ACGAGAAGGGTCCTCGCCTGGAGGCTGCATTTAGCGGCCGATTGGCCGCGGAAGAAAAAGCGTGCCGACCCCCCGGGGGT
GGTGCGCCACGGCAAGGATGATTGGCGACGCTTCGATGCGCGGCATCATGGAAAAAGTGCACAGTACAGGACCAAGAAAG
AACCGTATCTTGCTTTCTTCTCTCCAAATA

>UmT-165

GACTTGACTCAGCTGAGCAGCTTGAGCTTGGATCAGATTGTCGTTTCCCGCCTTCAGTTTAAACTATCAGTGTTTGAGCG
TATTACATGAAGAATGAGTTCATTTTCCTCGTTGAAATCGAAAAAGAGCGTTGATGCACTTTATCGCGTGATGCCTGCTG
TGCCGACGTACACCGCGAACGTTCTTCATTTGGCAAGATGTACTGGCCTCTACATCTCGCTCTCTCTGCTACTCCACTTA
GATGTCAAACCAGCTAGAACACCCGACTCTGCTGCTTCCGGGACCAATTGAATTCGATGATGCCGTACTTGAGTCTATGA
GCCACTACAGGTTTGGATCCGCTCCTTTTTAGTCTCTGCTTCTCCACTTATGACTTCGTCGTCCAGCCAGAGCCATGTCA
GCGCCCCATTTGTCGCCACATTCGGTGAGACCCTCACCATGCTCCGGAAGCTCTTTCAAACCGAGAATCCCTCGTCACAG

CCCTTCGTCGTCAGCGGCAGTGGCACACTTGGTTGGGACATGGTTGCCGCGAACCTGGCGGAGAGGGGCGATGATGTGCT
AGTGCTCCACACCGGCTACTTCGCCGACTCGTTCGCCGATTGCTTCGAGACCTACGGCGTCAATGCTACACAGCTCAAAG
CACCCATTGGTGATCGACCGCAGCTGCCAGAGGTTGAGAAGGCTTTGAAGGAGAAGAAGTACAAGCTCCTCACCGTTACG
CACGTGGATACTTCAACCGGTGTACTTAGTGAGATCAAGGCACTCTCGGAATTAGTGCACAGGGTTAGCCCAGAGACACT
AGTTATCGTGGACGGCGTCTGCAGTGTTGGTTGTGAAGAGATCCGCTTCGATGACTGGGGGCATCGACGTCGTCTTGACT
GCCAGCCAAAAAGCCATCGGCTGTCCCGCCGGTCTTTGCATAATGATGTGCTCCGGCCGCGCCATCGAGACGTTCAAAAA
CAGGAAAACGCCACCATCATCCTACTTCGCCTCCTTTCAAGAACTGGCTCCCGAGTACGTTATCACTCTCTCCACCATCC
TCGTATACCTCCGAGTCGAATCCTCACTAACAACACTTCTAGTAATGCAACCTACGACGCCAAAAAAGCGTCCTACTTCG
CCAGCCCATCCCCCAGAACTGATCCACGCCCTTACAACATCATCAGGCGAAATAAAAATCCGTTCGCCATCTCCAACCCG
TATGCCCGCCCACCCGGAACGCCTCGGAGCGGTCAAATCACCGGTGCGCCTCCCTCCGGCCTAAGCCTATGTTAGACCCA
CATAAGCTGCG

>UmT-166

TTGGCAAAGGTACCCATTAAGATGAGCGCTTGAGCTTGGATCAGATTGTCGTTTCCCGCCTTCAGTTTAAACTATCAGTG
TTTGAGAAGGGACTCCCCCTCTCCATTTCTCTGCTTCTCCACTAATTCCTCCACCTGTGACCAATCCCCTTCCTGTGAAA
TGTAGGAAGTAGGAGGTCGTACAATTTCGAGCAGCATCACGGTAGCATCTCCTGGGGCCGTCGCAATGTGGATAGCGATC
TGCTTCCAGGGCATTCTCGAAGAAATCGGAAAGAATCATATTCTCCGCCGCTATAAGTCCATGAACACCGACTTCACCCA
CGGTCGGAACCGTAGGGTTGTAGTTTGTTTATTCGAGACTCGATGTCCGTCCTCAGCACAGTGGCCCCCGGGAATAAGCA
GTAGGATACCGGCACCCACCGCATTACTCAAGGAGACCCAGATCTGACCTTCATCCCACCATTTCTTATACTTCTGATAC
TCTTGCTCTCTGTGATTCCACTAAAATGACTCCCTATATTTAGGATGAACTTCTCTTCATGGCTGTGTGAGTGCCCTTGT
GCATTGGTGTTCTATAAGATGTGGTAGGACCGATTATTCCCATCCCCCCCTGGGAAATTCATATCCACGATCCTACTACT
ACAAAACCATCTATTCGTCATCTCAAGCTTTTGATAAGCCAGACTCTGTGGCCCACTGGTCCGGAATACAGAATGCCACG
TTCACAACTCCAGGGCATGATCTCCATCGATTACGACGGACGGCTATAGCTCCCTGTTCCTCCAAACCGAAAAGGTCGGG
GATAGGCTCCCTTCATTCACAGCCAGGTTGACAAGATCTGCGATCGACTTGAAATGAATATGCCGGGACAATGACTGCCT
ATCATAGATGATATCTCCCATGCTACCGACGGACCTGCTTCATTATACTTTTTTTCTCTGTCTAATGGATTCCCTGGACA
GCCCCAATTTCCACTGCCCTTTCTCGAAATTCATGAAAAGTTACTAAGTTCAAGTGCGCAACCATTCGGCTCCAATTCCC
CTGGACATACTATGGTCTGGCAAATCAGCCTCCTGTAATTTCTTGCTGTTTTTACTTTGCCAACCCACCTGGTTGGCCT

>UmT-167

CTTGTTAAAAGGTATCGAGCATCTGAGCAGCTTGAGCTTGGATCAGATTGTCGTTTCCCGCCTTCAGTTTAAACTATCAG
TGTTTGA

TCTGCTTGTCCACTTATGTTGCGGCGTTCCGACTTTGCCGAGTTTGAGGGCATAAGCCCCACGCTAATGAGCGAGCGCCG
CTACTATGCTGAAACCTCACGTCACCAAATACAACGTCTTGAAACATTTGCGACCAGAACTCTCCTCCTTTTCTTCAAGC
AGCTACAATTGATTAGCGCTCATAAACCCATTTTCCGTGTCAATTATTCATCATCCCGAACGGCCAGATTTTAGTTTACC
CGTGCGATCTGTTCGCGGAGTTATTTGCCCGTTTTGTGGGAACATGGGTTGATATCCAGCCAAGCATCTTCAGATGCTGA
TGTTATGTGTTCAAGCATGTGTCTTGCGCTTGTAGATATGACATATAGTTGAGGCGGTGTTGTGTGTGGAAGGGGTGTCT
TTTCTCTACGTAATGCGTTCTTCGCTCTTCGTATTGCGTGCTTCCGATTCTCCTCACGTCGTAATCGATCCTTCTTCCAT
CTCCTCATCGAAGCGTATTTGTCTTTGCTTTCTCCTTTCGCTCTGCACAAGCTCCAGCAATTCTCAAACCGCCCAATCAC
TATGCATTTCTCCACCCTCCCCATCCTTGCCGCCTTCCTCATCTTCGCCCACCCTGCCGTCTCTGCTTCTCCACTTAACT

>UmT-168

TATGTCAAAGGATCATTCAGCTGAGCAGCTTGAGCTTGGATCAGATTGTCGTTTCCCGCCTTCAGTTTAAACTATCTCTG
TTTGACCATTAACAAACTGGGCGCACCCTCCAAATACCGCAACTGCTCTCCGATCCCCCTGACCAACTTCTCCAAATACG
GCCTATCGTTCCTGCTTCTCCACTTGCCATGCTCGCTCAGTGGCGGGAAAAGCGTCTTGTCTGGCGCGAAGTGGTTTAGG
AACGGTGTGTGCGCGGGTAGGGATTCGTTGAGCGTGGCATCAATGCAGATGCTTGTTTCGTAAGCCCAGAGCCGGGCAAC
GTTGCGGGGTGTATATCCACCGCCGCCGAGAACGAGGAGTGGGAGGTTGAAGGTTTTGACAAAGGAGACGCAGGCGCCAT
GGGCACGGATGTTGAGATTGAAGCAACCGAGTCGGTCGCAACCGAGGGAATCGGCGCTGCATTGGAGCACGATGGCCGTG
GGTTTGTAGTGCTCGACGCAGGGGCCGACGATGTCGCGGAAGAGACGGATATAGTCTTCGTCTTCGATGCCGTCATTTAG
GGGCACGTTGAGCGCATGATGCGCGCCCGGGTTGAGCGGGTTGAGCGGGCCAGTTGACGACAGCGGGCCGGTGCCGGGGA
AGAAGCCGTCTTTGTCATATTTGTGGAAGGAGAGGGTCATGACGCGGTCGGTGGACCAGAAGGCTTGTTCGACGCCGTCG

>UmT-169

CACGTCAAGGTTCAGCAGATGACGCTTGAGCTTGGATCAGATTGTCGTTTCCCGCCTTCAGTTTAAACTATCTCTGTTTG
ACGCCCCCTGTACCCAGGTGGGTACAGGAAAGAGCTCGAAATTTAAGGAATCGGTTGACATCATATTCTAACTCTTGTCC
TCCGAGATGCAGCGGCTGCTGCTGCTTGATCGTCCCCATCACTCTCATCTCAGCTCCACCACTAATAAAAAAGCTTGAAA
GAAGAAAGTTATGTAGCAGTCTGAGTTCTGCTGTATGCTTTTCTGCCTGACTTTTTATCTTAAGAACTCAGAAAGTTATT
AATTATGTAAATAAGTCTCAAGTTGTGATGTTCGGTCTTGATTTTCAGGGTCTTCTCACTATTCAGAGTCAGAACTTCCC
ACTTAAAGTCTTACAACACCTAAAACTTTAACACCTCAGGAGTTATGACAAAATGGTATATGAGAGAATTGTAGAGATCA
CTAAAGTGAGTAGTTCTTACACTGAAAGTAATATCATAGCTGAAGAACTCACTGTTGTACGAGATAGACAAGTGTGGGCG
TTGAACATGTTGATGTTCTTCCCTTTATACAGCACAGCGATCACAAAAAAGTTGAGTATATACACACTTACAACACACCT
AA

>UmT-170

CCGCAAGGCAGATGAGCAGCTTGAGCTTGGATCAGATTGTCGTTTCCCGCCTTCAGTTTAAACTATCAGTGTTTTGAGGC
GTTCTTGGCATTAAGATGAATGAGCAGGCTTGGGTTAATAAGTCATCCGAATATGTTACTACTAGTGGTATGTGGTTTTG
ACAGTATGGAATTCTTGTCAAGGCATTCTGTCAGGCCTGACAGAGGTCTGCCAACGATACGTTCCGTAGCATAGTTCAAT
AATACGCTTGATTTCTTATGGCAGTTTGTATGATCTCTCTTCTCCACTTACATGGCTGTCGTTTTGCAACCTGCTTCTCC
ACTTACCCTGGCCTTACTTTTACTACCTGTTCTTTTACTTTAGGTTGATTTTCTTAACGTCGCGGCTGGTATAACCCTGT
CACTGCCCTTCAACTCCGCAAGAAATAAATGTTCATTATTAATGTTTTTCTTGAAGTCTGCTGTGTCGGCCTCAAGTGGC
TTGGTCTCTTGGTCCCTGGGCTGGCCAACATCGAGGAACTTCTCAACACCATTGGGTCCCAGGTTGACGTTGGATGCAAA
GAAGTCACTGTCTTCCTCCTTGTATAGGGGACTGTCCACTAAGGGAGGCTCACCAACACCCGTCTGACCTTGATCTGCTT
TAATAAACTCTTGGCAAAACCGCGCACCGGGCAAGTGACACTGACAGAGTCCAGTGATCCGGCACCATCCCTAGGACTCT
AACCACCTCCTTCATCCACCGAAATTGTCTCCCAGCCGA

>UmT-261

TCCGAATGTAAGGCTAGAGCAGCTTGAGCTTGGATCAGATTGTCGTTTCCCGCCTTCAGTTTAAACTATCAGTGTTTGAA
CCGACGTACGCTCTCGGCCCTATGCACGTGATCGGGATCATCTGGCTGAAGTCGTTGCAGTTGTTTGGCCTGTCTCAAGA
GGAGATCGGAATGTCATTACTCGGAGTTGGCCGACCTGATCGAGTGTGTGCTCTAGCCCTTTCCTTGCTTCCTCGTTGCT
AACAAGGGTAGGGAGTCGCATCGCGCTCTCGGTATCGAAACGCCAAGGAAGAGACCCAAAGGGATTTCCGGTCCTACAGC
TCCTGCCATGGCTATTGTGTGTCTTTCCCAAGCTTGGATTCTGGCACCTGTAAACTAATTCACGCCAGCGACAAGATGGA
GGCAAGCCGAATGAACGGAGGAATTCGCGCTCGTCTACAGGATCATCGCCCCTATTACTTTCCAACGTCCCATACTCGCA
CCTCTCTGCTTTCTACACTAATCAAAGCCGAGCATCCATTCAGGTACGATAGTGGTCCAGCGCATGTTCATCGATGCTAA
TTGCACGACAAGCAACTACTGGACTCATCAAGGCGGTCTACCAGAAGTTATCGGGGTTCTGCCAAGCAAAGAACAGGCTG
ATATGTAAGTCTCTCTTGTTTCTTTGCTCGCATTCAAGAGTAGCTCGCATCAACTCTTTCAATTCTCTTCGCAGTCTTAG
CTCTCGTTGTCGTCCGGCTGTCTCGTGTAGCATGTTTTAACGTCCATTCCAGTCTCATAGCCAAGTACTTTGACGCCGTC
GATCCTGTATACTCTCTGTATCTCTCACATATACCCT

>UmT-262

CTGACATACTGAGAGCTTGAGCTTGGATCAGATTGTCGTTTCCCGCCTTCAGTTTAAACTATCAGTGTTTGATCTTTGTC
TTGCCATGGGTACCAGGTGTTGATCTGATGGACACTGATCGTGAGGTAGCTGACTCAGTATGAGAGGGAGTCGGAGATCT
GCTTGGAATATAGTATATACTATGATCTTCCGCGTCTTCTATGTATCGTTGGCTTTGATAAGTATACCTCTTTCTATCAA
ATACGTTGTCCTCCGATTTATGCCTCATGGTCCTGGAAATCAGACGGAGGTCTCGTCGTCTATGTTTCTCCACTTGATTG
CCGTCTCTATGAACGAGTCTACTTTCTTCGAAGAATTTCGATCCAGAGCATCCTCGATCAGAAGCATGTTCTGATCGAAC
GCGATTTTTCATCAAATATCTTGGTCTGTATGACCCAGGACTGGCTAGCTTCTCGCCGTTGAAGAATGCTTTGTAAGCGC
TTGAAGCCTCCGGAATTACTGTGATTTGTGATTCGCGAAAAGTCCGCTCGGTCAGTCTGAGCGGCTCCTCAGAGAACGCC
GCAGTATCCTCGTGCCTCGGAAGATCCTGATGTACATCGAAAGGGACTAGAGCAAGTTGAGCTTCCATGTACTGATTACT
ATGTTGCTTTTCTAGAAGTGATTGATTCAGCGGATAACTCATCACATCTGAGGGCTTTCTCCCTTCATTTTCGGAACCGA
AGTCTGAGTTCCCACTTTCAGAGGATCTGCCCGGGTGAGGGAGACGAAAACGTGTCTGACACTCTATGAGTCATCGTACA
CTATACACTAGTTTCGGTTCGCCCCGCTTTGTTTAGTACCTCAGTCGGTCGGGCTGAAGGCACACACGCCACAA

>UmT-263

CTAGAAGGACTGAGCGCTTGAGCTTGGATCAGATTGTCGTTTCCCGCCTTCAGTTTAAACTATCAGTGTTTGATCTTGTG
GGAGCTGGTGGTTGGACAGTCTGGCATCGCTCGTTGCCGCTACATGCCCGGTCCGCTGAGTCGCTGTGCTGCTCCGTCGC
TGCCAATGAGATGTCCGGGAAAGCGACGCAACTGATTCGTGCTCTCGCAAGAGACTGCGTGCATGGGCAGGCAGGCAGGC
AGGCGGGCAGATTGAGGCAGGGGATCGCTTCTCCTGCATCTTGCAGCTGCACTTTTGGATTCCGTGCAGGGCCGTGGCCA
TGCCTCGGATACTTATCGGCAGAACAGCCAGAGGCTCCCATCTGCCAGCATCATATCTACGGCATGTCGTCGGCACTCTA
CATTGCTCACCGTTATCCCGTGCTCCGATACTTCCTCCATCGTCACAATCATAAAGCCATTATTCATTGTTTGGCGGCGA
TATCGAACTGCCATAAGGTTCAAGCTACACTGAAGCTTCGGCCGAAGTAGACTTCCATTCCAAACAGACCAGGCTTCTGG
AAGTTGTCCAGACGACTGTTGCCGAACATTGATTAATCGCGGACACAGCTAACCCTTCAGTTCCAGTAGCTACCTTACCT
TATGTCGCTCCGACTCCTCGCACACTGTCGTTGGATGTGGATGACTTCGAATGTATCCAGTGATGAAAACGGGTGCGTTC
GCAGCTTGCGCTTGACAGCTCATCCTCACCACCTTGCCGGGTTTCTTCTGCGCACCCCGCCCCCCCCCCCCACAAAAAA
AAAAAAAACCTA

>UmT-264

CTTTAATATGAGCGCTTGAGCTTGGATCAGATTGTCGTTTCCCGCCTTCAGTTTAAACTATCAGTGTTTGAGCTATTCAT
GCCATTCTCTTTGCCGTCCGAATAATAATCATCCATCACCTTCGCTTCTGCAGCATGGCGTCTCCTGTCGAGCTTTCATC
TCCCGTTCTCTTTTTAGGTGGGGGGACCTGGGGTGTTTCCACCTCTTTGCATCTGGCGCGTCGCGGCTATGATAATATTG
GCGTTCTTATGTCATACCCTATACCCTCTGCCTGAATTGTGCGCATGATTTTTGAGGTTATGGGGGAGCCCGGGACATAG
CCTAGTATTTGACTTAGGAGATTACACAACTCCTTTTGACCTTCCTGAATCACGCTCATATGCCGATAGCGAACATCATT
AATCACCTTGATCTTATTCTCGTCGAATCCTGTTGGTGGCGGGCTCACAAGGCTGGATTCACAACTTTGTGTTCAAAATC
GACTATCCTGATAGGGGCTATATCGTTGCTGCTTTCTCCGAGGCCGCCATTGTTTGATACAATGCCGGGAGGAAAAAACC
ACACAACACTGATGTTGCTATATAATCATCACCCGAAGAGTTCCGTAAGACTATGCCTGCAGGAGTATTATCACGATACT
TTCCCGGCTGGCCACGCTTCTTCAAGAAGACTGGTGCATGATGGGTGCACGCCCAGAGATGAATGACTCTCAGCCGTCAC
AGATTGCACTAGACTTGGTCGTCATCTTTGTGACTGGCCTGCTTCGACTGAAGCGATACCGTTCAGTGGTTATCCTAAAG
GATAGAAAGACGTGAGGGGGGCGCGTACTGCCAGACGGCAGCAGCATAGAGCAGAGACATTCTTCTGTGCAGAGCACAGC
AACCAGCTTTCTAG

>UmT-265

GCAAAAGGTTCCCCATACTATAGCGCTTGAGCTTGGATCAGATTGTCGTTTCCCGCCTTCAGTTTAAACTATCAGTGTTT
GATAACTAATTATAAATATATTAGCATATTGAATAACTATTGCGCTTTTATACTTATAATATAACTTTCAGTTTATAAGT
TGTAGAGAATAAGCTGTTTAAGAGTTTTCTTATACAAAGTTTCTTTTTATGTATTGTTGTTGTTGAGTTATTCAAAAAGT
CATTAAAAATCTGTTTATTAATGTTGTAATTTTCATCACTGAAAGTTTGAAATCAATGTAACTTTTTAACAACAACAGTT
ATAAAAAAACTTTAAGTTATAGAACTGTTTAATAGCTTCTTCTCTACAACTTATAAACTGAAAGTTATATCATAAGTATA
AAAGCGCAATAGTTATTCAAAGTATGTGACTTTTTTTGAAAGATTTTTTGCTATTGAAAGCTTGAGTTAAATATAAGTTA
ACAACAACAGCAGTTATGAAGAAACTTTTAATTTAACATTAATAAAGAATACTGTTTTTTATAACTTATGCATTGAAAGT
TTCTTCATATCTGTTGAGGTGCAAAAGTTATTAAAGTGTGTAAATTTTTAAGTGATGAAAATCTTGAAGTTCTTTAAATA
TTCAGAACTCAACACTTATACAGTGCTTTCAGACTCAACAAATCATGATTTTTTTATTGCAGAAAAGTGAATTTTAGCAA
AAGTTTACTTATTATAAGTTTTACTGTTATTATAACACTGTGTGTGTACTTGTTTAAACAATTGTTTTAATAATTGTTTT
TTATCACAAAATTTTGATGTTCTGCTCAGATTTAGTTGAGCTCAAAGCGCTAGAGTGCTAGAGCGGCTGTTAACTGAGAA
CATGGAGAATCAGAAAGCT

>UmT-266

CTTTAATATGAGCGCTTGAGCTTGGATCAGATTGTCGTTTCCCGCCTTCAGTTTAAACTATCAGTGTTTGACTCGGAAAA
TCACGCCGTAAGTAAGAGAATCGGACATAAGTCATGCATATAGAGAGACCCCCCGGGCAAATCTCTTAATGAGCTCTTGT
GAATTTCTAATTATCACTACAAGTGGCAATTTCCAGCTGAGGGCCCTCCTGATATCACAACAGCAAAAGTGATATATTTA
AGTCCTTGAAAGGTAAGTGGAAGAGATTCACGGCTTACTATCACTGCATCAACTGATACTAGATATCTGTCTTGATTGTT
GAGAGGTTGAGGGGCCCCTCAGCTGAGGGTCAGCAGTTCTCTCTCTATGCATGACTTATGTCTGATTCTCTTACCAACCC
ACCCCAAGTAACCGAACGAAAACCCAACTGTATTGCACGAGCCAGACCCCCCACTTTTACCAACTCCGCAAGACAAAACC
ACATCCCGACGCTTGAACCACCTTGCAGACCTCACTGACCATCACCCTCCACTCCCTCAAGCCCAGATCACGTCTGCACC
TTATTATCCGCCCCAACCGCCTGCTCATAACTCGGCAACACCGCGCCATTCCTACTCCCCCCCGCCTGCGCCTGCTGGCC
CCTCTCCCCCTGCCTCCCAACCCGCATACTCTCCCCAGTAGTGCCATACCCGCCATGCTCCGCTTTCTCTCCCACTACTA
AGGTG

>UmT-267

CATAATAAGATGAGCGCTTGAGCTTGGATCAGATTGTCGTTTCCCGCCTTCAGTTTAAACTATCAGTGTTTGATTACTCC
ATTCAATCCTTAGCGCCACTGTCGAGTCTCGAGCCGTATCCGCTACACCAATCCGGACTTGCCGCCCCGCACACTCTTCC
GCCCCTGCAACCTCAACATGCAGCAGCTCTCTCTCCCGCCCATGACCTGTTCGGCCAGAATAGACAACCCCCTCGATCTT
CACAACCCAGCAATGTCTCTGCTTCTCCACATTAAATGGGCTCCTATGTCCATTATGCACCTCAACCCTCGACCCGATTG
AACGGATCACAGCCTTCTTACATCCAGCCGGAAAAATCCTATCGAAGCTCTCAAAACTACATCCCGCAGTTCTCAAACCC
TCCATACTACAACCGAAATGTTGTGACGATGGCGCCGCAAAGCCGAATGCCCGACCTACGGGCTATGCCCGCAGGGAGGT
CTACACCAAGCATCAATGATGTCCTGATCCCAATCTCAGACTCAGGGACTACTCACCACCTTCCTAATCACCCAATTAAG
ATTGCCATCGACGCATGTGGTAGGCTCTGAGAGGTTGGAAGGAGATACTCTCGATTGCTGCTGCTTCTGCCACTTACGGA
TGCTGGTGAAGGATAAGCAAGCGCACAGCCCGCTGTGATATCGATAAAGGATGCAGAATGGAAATTCCCTTGTCCACATT
GTAGCAAAACCTACCTGCATGCCAAGCATCTTAAGCGGCATCTTTTGCGGCGTAAGTTTCACATGAGCTCTCTTTTTAAC
TTACTGACAGCTACAGATACTGTGATCGACGTACTGTGTATACAACGTAAAGAATACTTTCTCTCGAAAGCGTATATACT
TAACCGGCCCATTTCGGA

>UmT-269

GCAAACCCATTTCACCTTCTGAGCAGCTTGAGCTTGGATCAGATTGTCGTTTCCCGCCTTCAGTTTAAACTATCAGTGTT
TGAGTGAATTCTCTTCGCCATCAAGGCTAACAAAGCTGCCTCGACGAATGTGGTTTGCGCCCGCGAAGCTGCTTCCGAAT
ATGCTCACTCCTCTGCCAAAGGCCCCTGGCACCGGGCGAACAGCATGGGCACTGAAGGGGGTAGAGGGCGTTCCAAAAGA
ATGTCTAACTTGGCGTGCCTTTTCAACGGCGCTCTCTGGTAAAGGTACTGCTGCAGCAGAAGCCACGTTATTGGAACGCT
TGCAAGGCGTATCCGGCATCTGAGCTTTACTCTCACGTAGTCCAGGTTGTATACTGTCTCTGTTTCTCCACTTAATGTCA
TCGGCCTCTACATTGCTCACCGCTATCCCGTGGGCCTATACTTCCTCCATCTGTCAATCATAAAGCCATTATTCATTGTT
TGGAAGCGATATCGAGACTGCCATAAGGTTCAAGCTACACTGAAGCTTCGGCCGAACTAGACTTCCATTCCAAGAATACC
AGAATTCTGGAAGTTGTCCAGACGACTGTTGCCGGACATTGATTAATCGCGGACACAGCTAACCCTTCAGACCCAGTAGC
TACCATACCTGATGTCCCTCCCACTCCTCGCACACTGTCGTTGGGATGTGGATGACTTCCGAATGTATTCCAGTCATGAA
TAACGGGGAGCGATACGCCAGCTTGCGCTTGACAGCTCATACCTCACCCACCTTGGCCAGGGATTACTTTCTGCTGCTAC
ACTCGTCTCATGCGTACTCTACTAAACAAGAGCACATACGTGACTGCGCCCCTCTGCGTTAGCTGCTTGATCCATGGGGG
TGCGAACTCTGTCTAGGGACCC

>UmT-270

CATTAAGGATGAGCGCTTGAGCTTGGATCAGATTGTCGTTTCCCGCCTTCAGTTTAAACTATCAGTGTTTGATTCTTCAA
AGTCTCTGCTTCTCCACATAACGGACGGGCACAGCTCCGACGCATACAAAAACTCTACCAAAAAGACAGGCGTCAGCCTT
CAGCCCTTCCAAACGACAGTTCATATTTGTAAAGACAACGACCACCAGGGTTTGTGACTGGTGAACATTCATTACTGTGT
ACATTCCATGCTCTTTTTGGCTTTAGAGCATATGTCGAAAGAATGGAGTATCTGTTGTACGTAGATTCGACTCTGAATGC
AGCGATCATCACCTCCATTACCCCTGCATGTTCCTGCTTCTCCACTTTCATATCTACCGCATGTCAACGGCACTCTACAT
TGCTCACCGTTATCCCGTGCTCCGATGCTTCCTCTATCGTTTTTATCATAAAGCCATTATTCATTGATTGGTGGCGAGAT
CGAACTGCCATCCGTTCAAGCTACACTGAAGCTTCAGTCGAAGTAAACTTCCATTCCAAACAGACCAGGCTTCTGGAAAT
TGTCCAGACGACTGCTGCTGAATATTGATTAATCGCGGACACAGCTAACCCTTCAGTTCCAGTAGCTACCTTACCTGTAT
GTCGTTCCGACTCCTCGTACACTGTCGTTGGATGTGTATGACTTCGAATGTATCCATTGATGAGAACTGGTGCGTTCGTA
TCCTGCGCTTGACATCTCATCCTCACCACCTTGCCAGGAATCTTCTGCGGACTACGTTGTACTGTCGCCTTGTATCTTGT
ACTATCTCTTAATAGAAGTATATGGT

>UmT-272

CAGTAATATGAGCGCTTGAGCTTGGATCAGATTGTCGTTTCCCGCCTTCAGTTTAAACTATCAGTGTTTGAGAGACGCTG
TCGAACTTTTCGATCAGAAACTTCTCGACAGACGTCGCGGTGAGTTCAGGCTTTTTCATTTGGATGCTTGGGTAGAATAG
GTAAGTCAGATTGAATCTGAAATAAAGGGAGGAAGGGCGAACTTAAGAAGGTATGACCGGGTCGTCCACTTACCTTGCTT
GACAAACGCACCAAGTTATCGTGCACCAAGCAGCAGATGATAATAATGTCCTCGTTCCTCTCTGTACTCCACTAAATGCC
GCCCTGCCCTTTTCCTGGGGTTTTCTTGTCGCGGATCTGGTCGCATAAAGTATTTAACTTGCGACTTCCCGCTTTACCCT
CGACCTCCCAACAGTCTGCTCTCTGCTTCTCCACTAAATTCATGAAATGAATGACATTCGTTACAATAAATCCCCCTTTT
TCTCCTTTCCAAGCACCTGGCACCCTACCTGAGAGAAACAAACATCTTCTGCGCTCTCTGCTTCTCCACTAACCTTAATG
CTGCCCGCACGACTGGTTTCCAGACTGGATAGCTGGCAGTGATCGCTAACCAATTAATGTGAGTTAGCTCACTCATTAAG

CACCCCACGATTTACCCTTTATGCTTCCTGGACGTGTGTGACATCGCAACTATCAGGGATGAAAGTAGCTGCACATACAG
CTTGCGCTTGACAGCTCATCCTCACCACCGTGCCAGGATCTCTGCTGCACTTGTCTCCTCTATATCACAAAAAAAAAATG
AA

>UmT-400

GGTAAAACGCACTACACCCTATGACGCTTGAGCTTGGATCAGATTGTCGTTTCCCGCCTTCAGTTTAAACTATCAGTGTT
TGATCAAACAAACCGTCCCCTTCCTCTATCGATTCTCTTGATAAGTAGCTCTCCTCTAGGTCGTCTTCCTGCTCTTCGTC
ATCAAGTCCTTCACTTCCTTCGTCTCTGCTTCTCCACTTTCAGGGCAGTATGGAGCACGAAAAGGAGGTCTATATTGAAT
AATTAACTAATTAATTATGGTATGAAGCCTGCATTTGCTCAGTTGCTAGCGTAGTTCAGTGACTGAGCAAATATGCTCAG
TCGCTGAGCATATTTAACTTTTGAACTCGCCTACAGTCACATATATGGTTAGGGCGGTGTTTTGACCTCTCGCGAAAACC
CTTGCTGCATATGCTCAAAAACAATACAGGGGTGGCTAAATCCCTCTACTAAGCAGCTCGCAAGCGAGCTGAATGATATA
TGCGTCGTAGAGTCTGTTCCAAGAAGCACAATCAGATCATTAAGCCTCGGGGGCTCAGTGATGTGGTTGAGGAAGTCACC
CTATCAATGTGAGATCCGTAATAGAAGTCCCCTCCCGCAGGATTTGATGACCATCGACGTCGGCGACGTGTACTATACCA
TCTCTCCCGTGGGAGGACCTAGGCCTTGAACAGGGTCTCGCCGTTAGGAACAGGGAAATGAGAGAAAGAAAGATCGAGAG
TAGAAAGTCAAGGTCGAGGTTCGACGTTGCGGTAGCGAGTCATGAGCGGGCGTTGGTGAAGCGGACTTTGATTTCAACCG
CAACGCGACTTCCCGTAGCTCCCATCCCCTCCCTTATCGCCATCTC
